# Regulatory cascade of NdpR and NdpR2 in controlling nicotine degradation operons in *Sphingomonas melonis* TY

**DOI:** 10.1128/aem.01130-26

**Published:** 2026-08-12

**Authors:** Qi Tang, Ziliang He, Shihan Wang, Yiming Cheng, Haixia Wang, Weihong Zhong

**Affiliations:** 1 College of Biotechnology and Bioengineering, Zhejiang University of Technology 12624, Hangzhou, China; Michigan State University, East Lansing, Michigan, USA

**Keywords:** nicotine degradation, regulatory cascade, TetR-family repressor, *Sphingomonas melonis *TY

## Abstract

In *Sphingomonas melonis* TY, the TetR-family NdpR and AraC-family NdpR2 coregulate nicotine catabolism. Despite overlapping target promoters, their hierarchical relationship remained unclear. Here, we elucidate a multi-layered cascade regulatory network where NdpR directly binds to the *ndpR2* promoter and represses its transcription. Structural and mutational analyses elucidate the structure-function relationship of NdpR, featuring a wide HTH span that supports a strong steric barrier mechanism on core promoters. The intermediate 2,5-dihydroxypyridine (2,5-DHP) acts as an effector, preventing NdpR from binding the *ndpR2* promoter. Furthermore, molecular simulations demonstrate a direct NdpR-NdpR2 physical interaction. We propose a double-lock regulatory mechanism operating synergistically at the transcriptional and protein levels. This precise dynamic regulatory balance enables cells to sensitively perceive environmental signals via basal metabolism and rapidly activate target gene expression upon signal confirmation, thereby achieving a balance between energy economy and efficient substrate degradation. Based on this cascade, we constructed a multi-factor system in *Pseudomonas* sp. JY-Q exhibiting bidirectional, reversible responses to nicotine, 2,5-DHP, and IPTG (isopropyl β-D-1-thiogalactopyranoside). This study uncovers microbial adaptive strategies and provides programmable, tunable regulatory tools for synthetic biology.

**IMPORTANCE:**

During microbial degradation of complex pollutants like nicotine, substrate uptake, transport, and catabolism are typically coregulated by multiple factors. Understanding how this complex regulatory network prevents metabolic energy waste, rapidly responds to substance changes, and thereby achieves temporal control over related gene expression is crucial for the development of metabolic control elements. This study reveals the cascade regulatory logic of two regulatory proteins within the same metabolic pathway and the regulatory balance during metabolism. Through structural and functional analyses of NdpR, we elucidated the molecular mechanisms underlying its target sequence recognition, binding of the effector 2,5-DHP, and interaction with NdpR2, thereby clarifying the dynamic regulatory process by which this system responds to environmental changes. Finally, we applied this system in a heterologous Pseudomonas host to develop a programmable multi-factor gene circuit. This study deepens our mechanistic understanding of microbial adaptive strategies and provides a dynamically tunable regulatory tool for synthetic biology and metabolic engineering.

## INTRODUCTION

Microbial processes such as energy conservation, carbon and nitrogen metabolism, antibiotic resistance, stress responses, and virulence are intricately linked to gene expression regulation by regulatory proteins (1–4). To achieve effective regulation, microorganisms have evolved diverse transcriptional regulatory factors. Notably, proteins of the TetR, AraC, and LysR families collectively account for approximately 30% of all microbial transcriptional regulators (5–9). These regulators govern distinct physiological processes and frequently interact with one another to form hierarchical and cascading regulatory networks (10–14). Such regulatory cascades are ubiquitous in bacteria, enabling precise and dynamic responses to environmental signals and playing a crucial role in microbial survival and adaptation.

For example, TetR family transcriptional regulators typically rely on conserved DNA-binding domains to regulate target sequences, responding to effector signals through conformational changes. To date, the crystal structures of multiple TetR family proteins (such as MmfR (15), PhlH (16), and DarR (17)) have been resolved. Most of these regulators achieve DNA binding and dissociation by undergoing relatively flexible conformational movements upon binding to their specific effectors. Notably, highly specialized structural adaptations of proteins within the same family were found during evolution. For instance, NicR2 (18), which regulates nicotine degradation in *Pseudomonas putida* S16, shares functional similarities with NdpR in this study; however, their targeted DNA sequence and effector responses are entirely different. This finding suggests that when exploring novel regulatory factors, special attention must be paid to their unique structural characteristics and target matching mechanisms.

Beyond the interaction between transcription factors and DNA, protein-protein interactions (PPIs) play an indispensable role in preventing expression leakage and achieving stringent regulation. In *Shigella*, the AraC/XylS family protein MxiE and its co-factor IpgC specifically target the *virB* promoter to form a negative feedback loop, thereby downregulating downstream gene expression (19). In *Pseudomonas aeruginosa*, the AraC/XylS family transcriptional activator ExsA is responsible for activating the type III secretion system. However, its activity is strictly limited by interaction with the anti-activator ExsD; when ExsD binds to ExsA, ExsA can no longer bind to its specific DNA target (20, 21). These studies demonstrate that the deep coupling of transcriptional regulation and post-translational protein interaction is a common and highly efficient strategy for microorganisms to achieve rigorous metabolic control.

To date, different transcription factors and their regulatory mechanisms have been reported in three nicotine-degrading pathways (22). For the pyrrolidine pathway as in *Pseudomonas putida* S16, the TetR family regulator NicR2 represses transcription by binding to the P*_hsp_* and P*_spm_* promoters within the *nic2* and *spm* gene clusters, and this repression can be relieved by the nicotine intermediates 6-hydroxy-3-succinoylpyridine (HSP) and 3-succinoylpyridine (SP) (23, 24). In *Pseudomonas sp*. JY-Q, the NicR2 homologs NicR2A and NicR2Bs share 100% and 70% amino acid identity with NicR2 from strain S16 and similarly function as negative regulators of nicotine degradation (25). For the pyridine pathway as in *Arthrobacter nicotinovorans*, the activator PmfR regulates the *purU-mabO-folD* operon (26), while the repressor HdnoR controls transcription of the gene *6hdno* (27). For the variant of the pyridine and pyrrolidine pathway (VPP pathway), the TetR family protein NdpR (28) and the AraC family activator protein NdpR2 (29) in *Sphingomonas melonis* TY have been established to regulate nicotine metabolism, while ORFW_13_-1 and ORFW_13_-2 in *Shinella* sp. HZN7 were proposed to participate in nicotine-degrading regulation as a putative sensor-response pair, although their molecular mechanisms remain unclear (30).

Notably, the nicotine-degrading gene cluster *ndp* is co-regulated by NdpR and NdpR2 in *S. melonis* TY. The transcription of genes *npdA*, *ndpHFEGD,* and *ndpTB* is repressed by NdpR (28), whereas NdpR2 (29) activates their expression. Both NdpR and NdpR2 share common binding sites on corresponding promoters, P*_ndpA_*, P*_ndpH_*, and P*_ndpT_*. The compound 2,5-dihydroxypyridine (2,5-DHP) functions as an inducer for NdpR, alleviating its transcriptional repression on P*_ndpH_*, P*_ndpT_*, and P*_ndpA_*. Conversely, 2,5-DHP acts as a negative effector for NdpR2, inhibiting its transcriptional activation of the same promoters. Further analysis of fluorescence resonance energy transfer (FRET) supported the existence of a direct or proximal interaction between the two regulatory proteins (29). Although these findings reveal a complex regulatory network, it remained unclear whether NdpR and NdpR2 influence each other and what functional impact the NdpR-NdpR2 protein interaction may exert on the overall regulatory system.

Therefore, elucidating the NdpR-NdpR2 regulatory relationship is important not only for understanding the regulatory logic of nicotine degradation in *S. melonis* TY but also for evaluating whether this catabolism-associated regulatory system can be further adapted as a programmable regulatory module. In synthetic biology, increasing attention is being paid to the standardization and repurposing of natural regulatory elements for precise control of gene expression and metabolic pathways (31, 32). Regulatory factors from catabolic processes are valuable modular parts because they often respond to specific small molecules and can be used for the dynamic regulation of target genes (33). These studies suggest that mining catabolism-associated regulatory systems may provide useful elements for constructing programmable gene expression systems.

In this study, we systematically dissect the cascade regulatory mechanism between NdpR and NdpR2 at both the transcriptional and post-translational levels. Transcriptional level analysis verified the direct repression of *ndpR2* by NdpR. Combined with protein structure and mutational analysis, we further explored the recognition mechanism of NdpR for target promoters, the functions of key amino acid residues, and the mechanistic effects of 2,5-DHP and NdpR2 on NdpR. Based on these discoveries, we proposed a double-lock regulatory model between NdpR and NdpR2. Furthermore, we successfully applied this regulatory system in *Pseudomonas* sp. JY-Q (34) to construct a multi-effector gene regulation system with bidirectional, reversible, and dynamic response characteristics, providing novel programmable regulatory elements for synthetic biology.

## MATERIALS AND METHODS

### Chemicals and reagents

(S)-Nicotine (99%), 2,5-DHP, maleamic acid, maleic acid, and fumaric acid were purchased from Chemsky International Co., Ltd. (Shanghai, China). The compounds 6-hydroxynicotine (6HN), 6-hydroxypseudooxynicotine (6HPON), and HSP were prepared as described previously (35). The 2× Phanta UniFi Master Mix (high-fidelity DNA polymerase) was obtained from Vazyme Biotech Co., Ltd. (Nanning, China), which also supplied the restriction enzymes and ClonExpress Ultra One Step Cloning Kit used for plasmid construction. Antibiotics, isopropyl β-D-1-thiogalactopyranoside (IPTG), and other general reagents were procured from Beyotime Biotech Inc. (Shanghai, China). All reagents and solvents were of analytical or chromatographic grade and commercially available. Kits for bacterial genomic DNA extraction, plasmid extraction, gel extraction, and DNA purification were provided by Tiangen Biotech Co., Ltd. (Beijing, China).

### Bacterial strains, plasmids, media, and growth conditions

The bacterial strains and plasmids used in this study are listed in Table S1, and the primers are detailed in Table S2. Strain TY (collection number CGMCC 1.15791) and its plasmid transformants were cultured aerobically at 30°C in lysogeny broth (LB) or inorganic salt medium (ISM) supplemented with nicotine, as previously described (36). *Escherichia coli* strains were grown in LB broth, consisting of 10.0 g/L peptone, 5.0 g/L yeast extract, and 10.0 g/L NaCl (pH 7.0), at 37°C with shaking at 180 rpm.

### Sequence alignment and homology modeling

The gene sequences of NdpR and NdpR2 were obtained from the *Sphingomonas melonis* TY genome. Based on previous studies, NdpR functions as a homodimer, whereas NdpR2 is a monomer. The amino acid sequences of NdpR and NdpR2 were subjected to *de novo* modeling using AlphaFold2 (37) to predict the 3D structures of the NdpR homodimer and the NdpR2 monomer, respectively. The dimerization interface and thermodynamic parameters of NdpR were analyzed using the PDBePISA web server (https://www.ebi.ac.uk/pdbe/pisa/) (38). For modeling the target DNA structure, the DNA sequence (5′-TTTATACAAGCGGATAATTGATTG-3′, 24 nt) identified by DNase I footprinting assay was extended by adding 10 nt protective bases at both ends. The double-stranded three-dimensional structure of the target DNA was then generated using BIOVIA Discovery Studio Visualizer 2020 (https://discover.3ds.com/discovery-studio-visualizer-download) (39). All predicted proteins and DNA structures were subsequently energy-minimized with it to obtain stable, low-energy conformations. To validate the reliability of the protein models, the Protein Data Bank (PDB) (https://www.rcsb.org/) was searched for structurally resolved proteins with high sequence similarity to NdpR. Sequence alignment was performed using DNAMAN, and structural alignment was carried out with PyMOL (v3.10) (Pymol Molecular Graphics System; https://www.pymol.org/pymol/) (40).

### Molecular docking and interaction analysis

#### Regulator-effector docking

To investigate the interaction between the repressor and its effector, the 3D structure of 2,5-DHP (PubChem CID: 893) was retrieved from the PubChem database. Molecular docking of 2,5-DHP into the predicted ligand-binding pocket of the NdpR homodimer was executed using AutoDock (41). Docking poses were ranked based on their binding free energy (ΔG), and the conformation exhibiting the most favorable (lowest) energy was selected for further analysis. The volume and properties of the binding pocket were validated and quantified using the ProteinsPlus web server (https://proteins.plus) (42).

#### Protein-DNA docking

To model the transcriptional repression complex, protein-DNA docking was conducted using ZDOCK 3.0 (43). The NdpR homodimer and the 44-bp target DNA fragment (containing the P*_ndpR2_* motif) were defined as the receptor and the ligand, respectively. All docking parameters were maintained at default settings. The resulting poses were re-scored and ranked using ZRANK (44) to identify the most probable binding orientation.

#### Protein-protein docking

To elucidate the structural basis of the regulatory cascade, the physical interaction between the NdpR homodimer and the NdpR2 monomer was simulated using ZDOCK. The NdpR homodimer was designated as the receptor, while the NdpR2 monomer served as the ligand. This docking aimed to identify the dimerization or sequestration interface that facilitates the observed PPI.

#### Interaction analysis

For all selected small-molecule and macromolecular complexes, the molecular interfaces were analyzed to identify key residues involved in hydrogen bonding, hydrophobic contacts, and electrostatic interactions. All structural visualizations and interface mapping, including the identification of residues within the putative effector-binding or DNA-recognition sites, were performed using PyMOL Molecular Graphics System (v3.10) (40).

### Site-directed mutagenesis

The reporter plasmid pUC19-P*_ndpR2_-gfp* was constructed via Gibson Assembly (29), in which the P*_ndpR2_* promoter fragment was fused to the *gfp* reporter gene and assembled into a linearized pUC19 vector. This reporter plasmid was subsequently digested with *Pst* I and *Eco*R I. Using Gibson Assembly, the wild-type *ndpR* gene was inserted to generate the control plasmid pUC19-*ndpR*-P*_ndpR2_-gfp*.

Site-directed mutagenesis was performed using this control plasmid as the template. Inverse PCR was carried out with primers harboring the desired mutations, followed by *Dpn* I digestion and transformation into *Escherichia coli* DH5α. Mutant plasmids were confirmed by PCR and DNA sequencing.

### Construction of a multi-factor combinatorial regulatory system

Plasmid constructions were through Gibson Assembly. The pMMB67EH plasmid (NovoPro, V013726) was modified by replacing the gentamicin resistance gene and removing unnecessary sequences to generate the backbone pMGm. The P_lacI-_*lacI* and the fusion fragment P*_lac_-ndpR* were assembled into this vector to obtain pMT. The pMT plasmid was linearized, and the *gfp* and P*_ndpR2_-ndpR2*-terminator fragments were introduced to generate pMTF. The resulting plasmid was digested with *Stu* I and *Spe* I, and the P*_ndpR2_* or P*_ndpA_* fragments amplified from the TY genome were inserted upstream of *gfp* to construct pMTF-R2 and pMTF-A. Plasmids were transformed into *E. coli* DH5α and verified by PCR and sequencing.

Plasmids pMTF-R2 and pMTF-A were separately electroporated into JY-QΔ*Nic1-2* (unpublished). This mutant retains the ability to take up nicotine but lacks the major nicotine catabolic modules required for rapid nicotine degradation, allowing intracellular nicotine to serve as an input signal while largely avoiding rapid metabolic consumption of nicotine (34). Cells in the exponential phase were harvested and washed four times with sterile water, and 90 µL of the cell suspension was mixed with 50 ng of plasmid DNA. Electroporation was performed under standard conditions (1.8 kV, 4.0 ms), followed by recovery in LB at 30°C for 1 h and selection on antibiotic-containing plates. Positive clones were verified by PCR and sequencing.

### Fluorescent reporter assay

Fluorescence-based reporter assays were performed to evaluate the transcriptional repression activity. For all samples, aliquots were transferred to black-walled, clear-bottom 96-well plates, and OD_600_ and fluorescence intensity (excitation at 488 nm and emission at 520 nm) were measured using a microplate reader. Fluorescence values were normalized to OD_600_ to obtain the relative fluorescence units (RFU).

For *E. coli* DH5α strains harboring the indicated plasmids, overnight cultures were diluted (1:50) into fresh LB medium to an initial OD_600_ of 0.1 and grown at 37°C, 180 rpm to mid-exponential phase (OD_600_ 0.5). Protein expression was induced with 0.1 mM IPTG for 2 h prior to measurement. Repression activity (%) = [1 − (RFU/OD_600_ of the test strain)/(RFU/OD_600_ of the empty vector control strain)] × 100%. The strain harboring pUC19-*ndpR*-P*_ndpR2_-gfp* was used as the positive control (100% repression), and the strain harboring pUC19-P*_ndpR2_-gfp* as the negative control (0% repression).

For JY-QΔ*Nic1-2*-derived strains (JY-QΔ*Nic1-2*-R2 and JY-QΔ*Nic1-2*-A), the cells were cultured in LB medium supplemented with gentamicin (50 µg/mL) at an initial OD_600_ of 0.1 and grown at 30°C (180 rpm). Inducers were added either at inoculation or at approximately OD_600_ of 0.3 as required, and the cultures were harvested after 5 h for fluorescence measurement.

## RESULTS

### Transcriptional profiles of *ndpR* and *ndpR2* under different carbon sources

In our previous study, *ndpR* and *ndpR2* were identified as key regulators of nicotine catabolism. In this study, their transcriptional profiles were examined using nicotine or glucose as the sole carbon source. As shown in Fig. S1, the transcription levels of *ndpR* and *ndpR2* increased significantly during the exponential phase in the nicotine-containing medium, suggesting that nicotine strongly induces their expression and highlighting their role in nicotine metabolism. In contrast, in the control medium containing glucose and ammonium sulfate as the carbon and nitrogen sources, the expression levels of both genes remained consistently low across all growth stages. Additionally, the transcriptional level of *ndpR2* was consistently higher than that of *ndpR* during the mid-log, late-log, and stationary phases. Notably, both genes exhibited a distinct downward trend in transcription from the mid-log phase to the stationary phase.

### NdpR negatively regulates the expression of *ndpR2*

**Fig 1:**
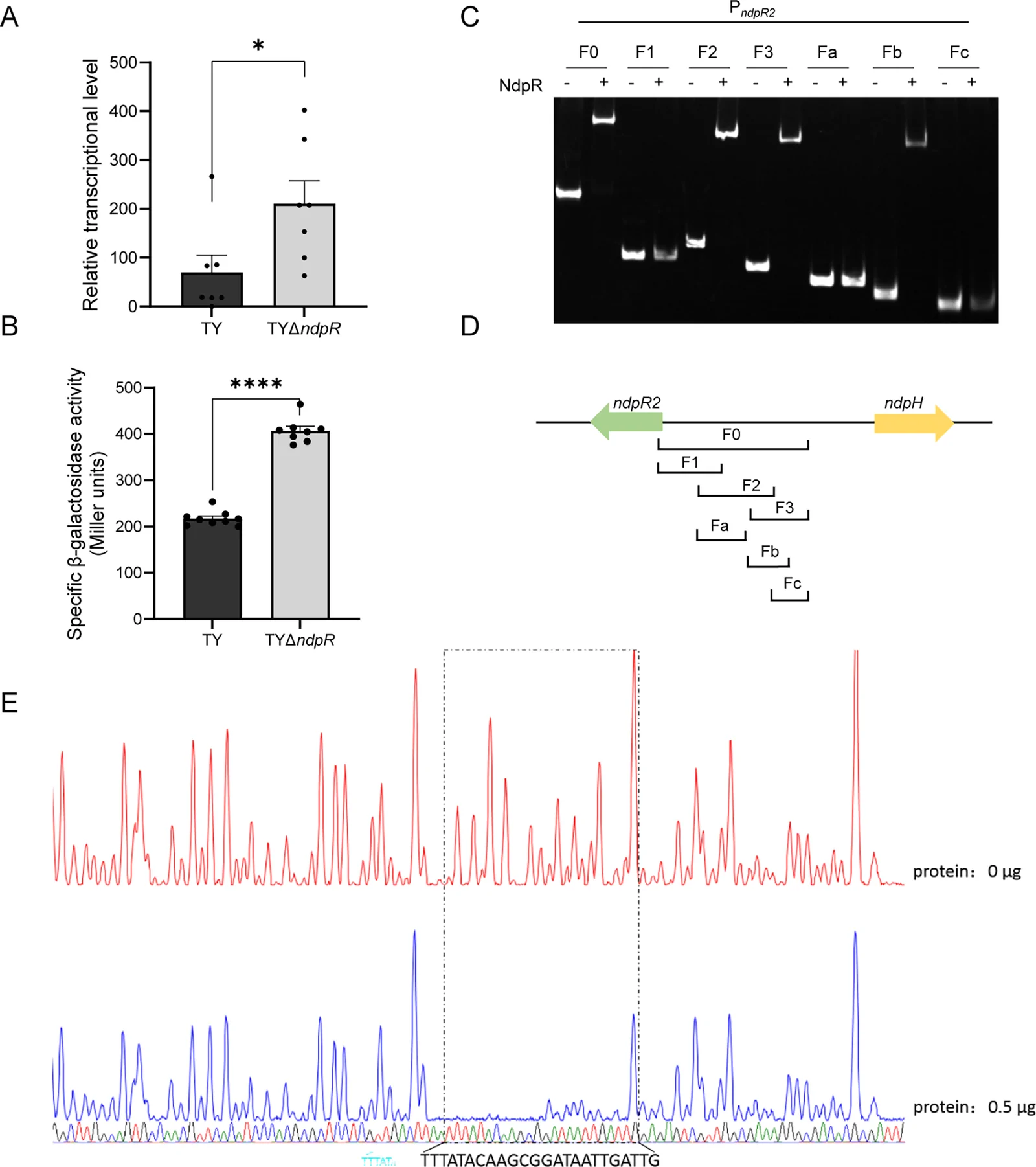
NdpR negatively regulates *ndpR2* expression and directly binds to its promoter P*_ndpR2_* region. (**A**) RT-qPCR analysis of *ndpR2* expression in wild-type TY and TYΔ*ndpR* during the mid-logarithmic phase when grown with nicotine as the sole carbon and nitrogen source. The 16S rRNA gene was used as an internal reference for normalization. The expression levels were calculated using the 2^−ΔΔCt^ method with three biological replicates. Error bars represent standard error. (**B**) Promoter activity of P*_ndpR2_* was evaluated using β-galactosidase assays. (**C**) EMSA analysis of P*_ndpR2_* fragments using purified NdpR protein. (**D**) Predicted binding site of NdpR within P*_ndpR2_*, with further subdivision of the promoter region to refine the binding site. (**E**) DNase I footprinting assay of the NdpR binding site in P*_ndpR2_*. A 6-carboxyfluorescein-labeled DNA probe was incubated with 0.5 μg of NdpR or without NdpR as a control. The region protected by NdpR is highlighted with a dashed box, and the corresponding protected sequence is displayed below. Statistical significance: * indicates *P* < 0.05, **** indicates *P* < 0.0001.

To investigate the relationship between *ndpR* and *ndpR2*, the transcriptional and translational levels of *ndpR2* were analyzed in both wild-type TY and the *ndpR* knockout mutant strain TYΔ*ndpR* (previously constructed in our study). Real-time quantitative polymerase chain reaction (RT-qPCR) revealed that the transcriptional level of *ndpR2* in TYΔ*ndpR* was three times higher than that in the wild-type TY (Fig. 1A). Similarly, promoter activity analysis demonstrated that the expression output of P*_ndpR2_* in TYΔ*ndpR* was two times higher than that in the wild-type TY (Fig. 1B). Consistently, *ndpR* complementation reduced P*_ndpR2_* promoter activity compared with the empty-vector control in TYΔ*ndpR* (Fig. S2). These findings indicate that in the presence of *ndpR*, the expression of *ndpR2* is downregulated, whereas in its absence, the expression of *ndpR2* is upregulated. Together, these results demonstrate that *ndpR* represses the expression of *ndpR2*.

### NdpR directly binds to the promoter region of *ndpR2*

To further investigate the regulatory role of *ndpR* on *ndpR2*, the *ndpR* gene was expressed in *E. coli* BL21(DE3) as previously described. To determine whether NdpR directly interacts with the promoter region of *ndpR2*, an electrophoretic mobility shift assay (EMSA) was conducted using the promoter region sequence of *ndpR2* and purified NdpR. The results showed that NdpR strongly bound to the promoter region (F0) of *ndpR2* (Fig. 1C). To determine the exact binding site of NdpR on P*_ndpR2_*, overlapping subcloned and sub-subcloned fragments of P*_ndpR2_* were tested for their binding affinity to NdpR (Fig. 1D). NdpR fully shifted the subclone F2 and F3 DNA fragments, as well as the sub-subclone Fb fragment of P*_ndpR2_* (Fig. 1C). These findings indicate that NdpR binds to the Fb region of P*_ndpR2_*.

A DNase I footprinting assay was then performed to identify the precise binding sequences of NdpR on P*_ndpR2_*. The results revealed that a DNA region (5′-TTTATACAAGCGGATAATTGATTG-3′, 24 nt), located within the Fb fragment of P*_ndpR2_*, was protected by NdpR (Fig. 1E). This binding site spans the −3 to −26 region relative to the transcriptional start site (TSS) of P*_ndpR2_*, completely covering the −10 box.

### 2,5-DHP acts as a ligand to dissociate NdpR binding to P*_ndpR2_*

**Fig 2:**
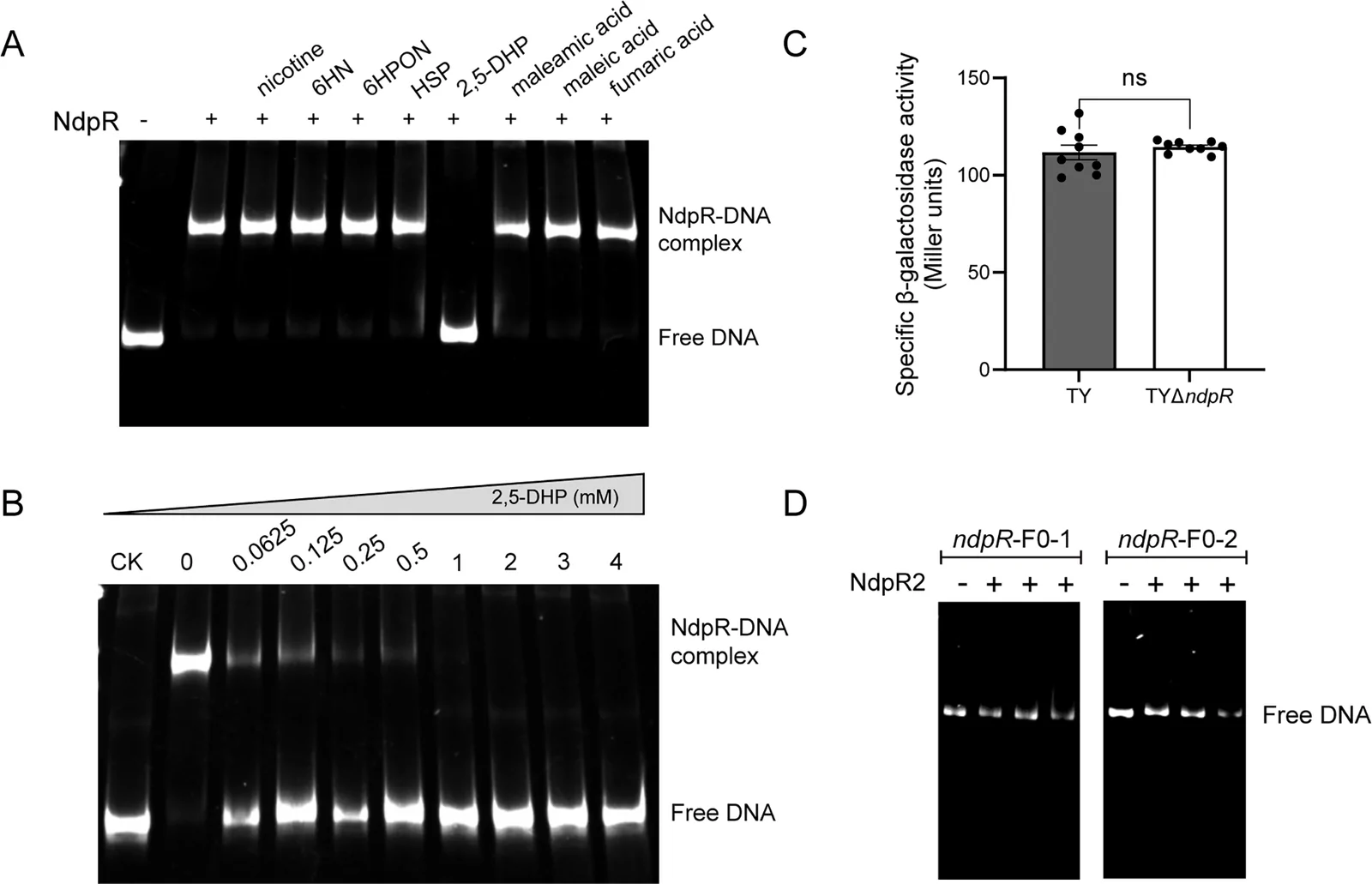
Effects of nicotine and its intermediate metabolites on NdpR binding to the P*_ndpR2_* promoter, and NdpR2 had no effect on the expression of *ndpR*. (**A**) EMSA analysis of the promoter sequence DNA (F0 fragment, 10 nM) with purified NdpR (200 nM) in the presence of 2 mM nicotine, 6HN, 6HPON, HSP, 2,5-DHP, maleamic acid, maleic acid, or fumaric acid. (**B**) EMSA analysis of the promoter sequence DNA (F0 fragment, 10 nM) with purified NdpR (200 nM) at varying concentrations of 2,5-DHP. “CK” represents the control group without the addition of 200 nM NdpR. (**C**) Promoter activity of P*_ndpR_* was determined using a β-galactosidase assay in wild-type TY and TYΔ*ndpR2*. “ns” indicates no significance, *P* > 0.05. (**D**) EMSA analysis of P*_ndpR_* fragments using purified NdpR2 protein.

The effects of nicotine and its intermediate metabolites on NdpR binding to the promoter region of *ndpR2* were analyzed using EMSA. As shown in Fig. 2A, 2,5-DHP inhibited NdpR binding to the *ndpR2* promoter regions, whereas nicotine, 6HN, 6HPON, HSP, maleamic acid, maleic acid, and fumaric acid did not disrupt this binding. Further EMSA analysis results demonstrated that increasing concentrations of 2,5-DHP resulted in a progressive reduction of the retarded complex, with complete dissociation observed at concentrations of 1 mM or higher (Fig. 2B). These findings indicate that 2,5-DHP acts as a ligand for NdpR, effectively dissociating its binding to the *ndpR2* promoter regions.

### NdpR2 had no effect on the expression of *ndpR*

To determine whether *ndpR2* influences the expression of *ndpR*, the promoter activity of P*_ndpR_* was measured in both the wild-type TY strain and the *ndpR2* knockout strain TYΔ*ndpR2*. The results showed no significant difference in *ndpR* expression between TYΔ*ndpR2* and the wild-type TY (Fig. 2C). Furthermore, *in vitro* EMSA experiments using purified NdpR2 protein and the promoter sequence of *ndpR* revealed that NdpR2 does not bind to P*_ndpR_* (Fig. 2D).

### Structural characterization and comparative analysis of NdpR

Previous glutaraldehyde cross-linking assays indicated that NdpR functions as a homodimer in solution (28), whereas NdpR2 remains monomeric (29). To investigate the structural basis for these observations, *de novo* structural models were generated using AlphaFold2 (Fig. S3).

**Fig 3:**
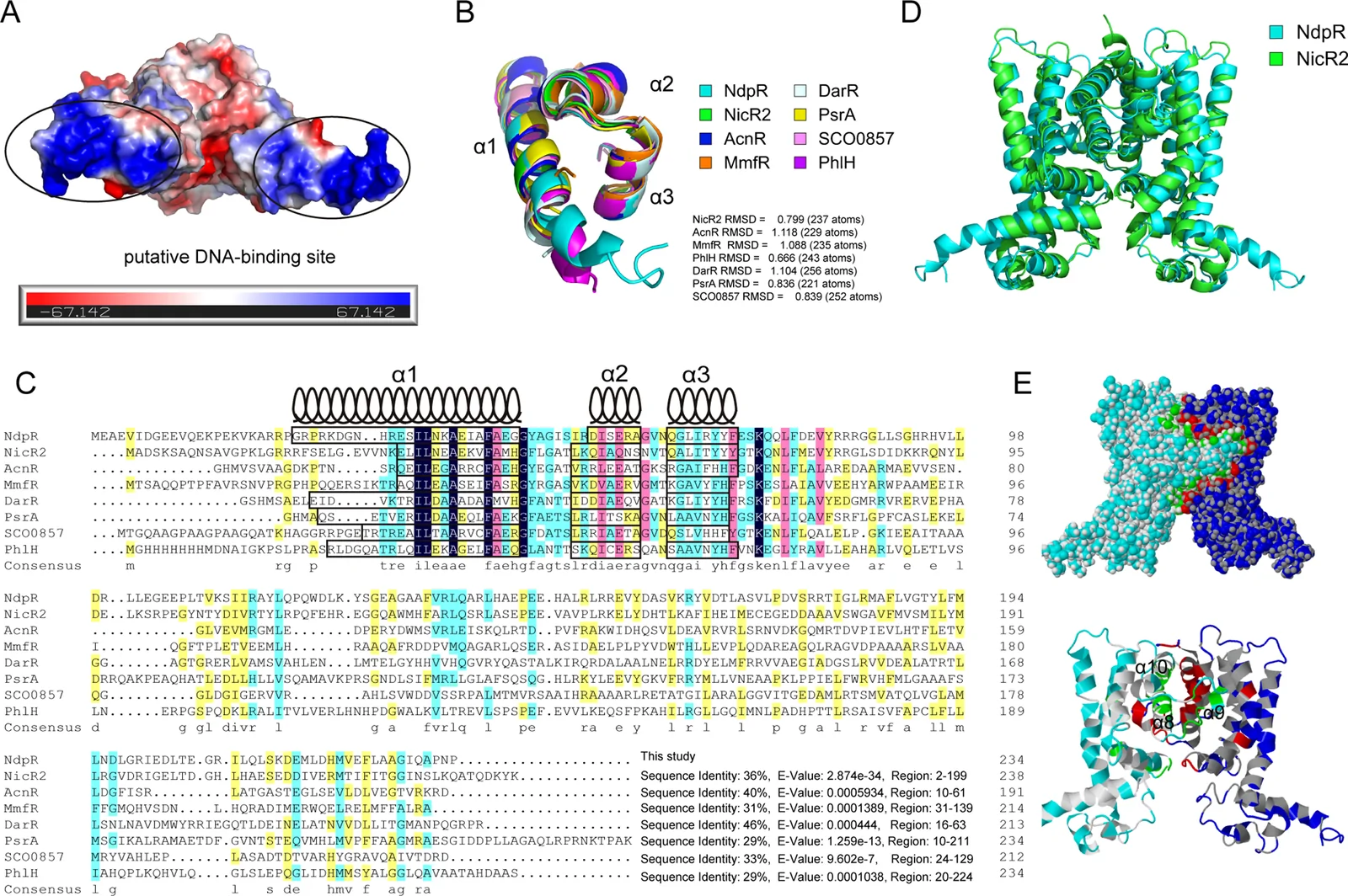
Structural characterization and sequence analysis of the NdpR homodimer. (**A**) Electrostatic surface potential of the NdpR homodimer. Positively and negatively charged surfaces are shown in blue and red, respectively (scale bar indicates potential in *k_B_ T/e*). Black ovals delineate the putative DNA-binding sites. (**B**) Structural comparison of the DBDs among TetR family repressors. NdpR (cyan) served as the reference structure for alignment. Helices α1–α3, comprising the conserved helix-turn-helix motif, are indicated. Local RMSD values and the number of aligned atoms for each homolog relative to NdpR are listed. PDB IDs: NicR2 (5GFL), AcnR (4AF5), MmfR (7KY1), DarR (8SV6), PsrA (2FBQ), SCO0857 (2NP3), and PhlH (7E1L). (**C**) Multiple sequence alignment of NdpR and its homologs. Secondary structure elements (helices α1–α3) derived from the NdpR crystal structure are displayed above the alignment. Sequence conservation is color-coded: dark blue (100% identity), pink (75%), light blue (50%), and yellow (33%). Sequence identity and E-values relative to NdpR are summarized in the right-hand panel. Crystal structure of TetR-family regulator (SCO0857) from *Streptomyces coelicolor* A3. (**D**) Structural superposition of the NdpR (cyan) and NicR2 (green; PDB ID: 5GFL) homodimers. The two structures were aligned with a root-mean-square deviation (RMSD) of 2.032 Å over 2,418 atoms. (**E**) Dimerization interface of the NdpR homodimer. Space-fill model with individual monomers colored light and dark blue. Residues participating in inter-monomer interface contacts are highlighted in red and green. (Bottom) Ribbon representation identifying helices α8, α9, and α10 as the primary structural elements involved in homodimer formation.

The NdpR monomer is composed of 10 α-helices organized into two functional modules: an N-terminal DNA-binding domain (DBD; helices α1–α3) and a C-terminal ligand-binding domain (LBD; helices α4–α10). The DBD adopts a prototypical helix-turn-helix (HTH) architecture characterized by a three-helix bundle (45). Electrostatic surface potential analysis of the dimer revealed distinct positively charged patches at the DBDs (Fig. 3A), consistent with putative DNA-binding interfaces. Structural comparison (Fig. 3B) and multiple sequence alignment (Fig. 3C) confirmed that sequence conservation is primarily localized within this DBD region across TetR family homologs, including NicR2, AcnR, MmfR, DarR, PsrA, SCO0857, and PhlH (15–18, 46, 47). Notably, the HTH framework within the DBD of TetR-family homologs is structurally conserved, particularly the α2 and α3 helices. However, this conserved secondary structural scaffold harbors a number of variable residues, and the difference in the length of the α1 helix in this region is also noteworthy. These features are likely to determine the specificity of DNA recognition among different TetR-family members. In particular, compared with other family proteins, NdpR contains variable residues within the α2 and α3 helices, and its α1 helix in NdpR is significantly greater than that of its homologs (Fig. 3B and C), suggesting that the NdpR homodimer may possess a broader spatial span for promoter recognition.

A Protein Data Bank (PDB) search identified *Pseudomonas putida* S16 NicR2 (PDB ID: 5GFL) (18) as the closest structural homolog to NdpR, sharing 36% sequence identity over the aligned region (E-value: 2.874e^−34^). Both NdpR and NicR2 primarily exist as homodimers. Structural comparison between them yielded a global RMSD of 2.032 Å (Fig. 3D). The LBD is typically highly variable among TetR regulators; however, the NdpR LBD aligned with the NicR2 LBD with a local RMSD of 1.943 Å over 1,845 atoms. These RMSD values indicate high structural similarity between the NdpR and NicR2 homodimers, supporting the possibility that NdpR may share a similar overall folding architecture and effector-recognition mode with NicR2.

Analysis of the NdpR dimerization interface (Fig. 3E) revealed that homodimer stability is mediated by an extensive interaction network involving helices α8, α9, and α10. This interface is stabilized by 18 hydrogen bonds and 28 salt bridges (Table S3). Thermodynamic analysis yielded a solvation free energy gain (Δ^i^G) of −22.3 kcal/mol upon dimer formation. The associated Δ^i^G *P* value of 0.245 (<0.5) further corroborates that this interface is highly specific and biologically relevant.

### Structural basis for P*_ndpR2_* recognition and DNA-binding mode of NdpR

**Fig 4:**
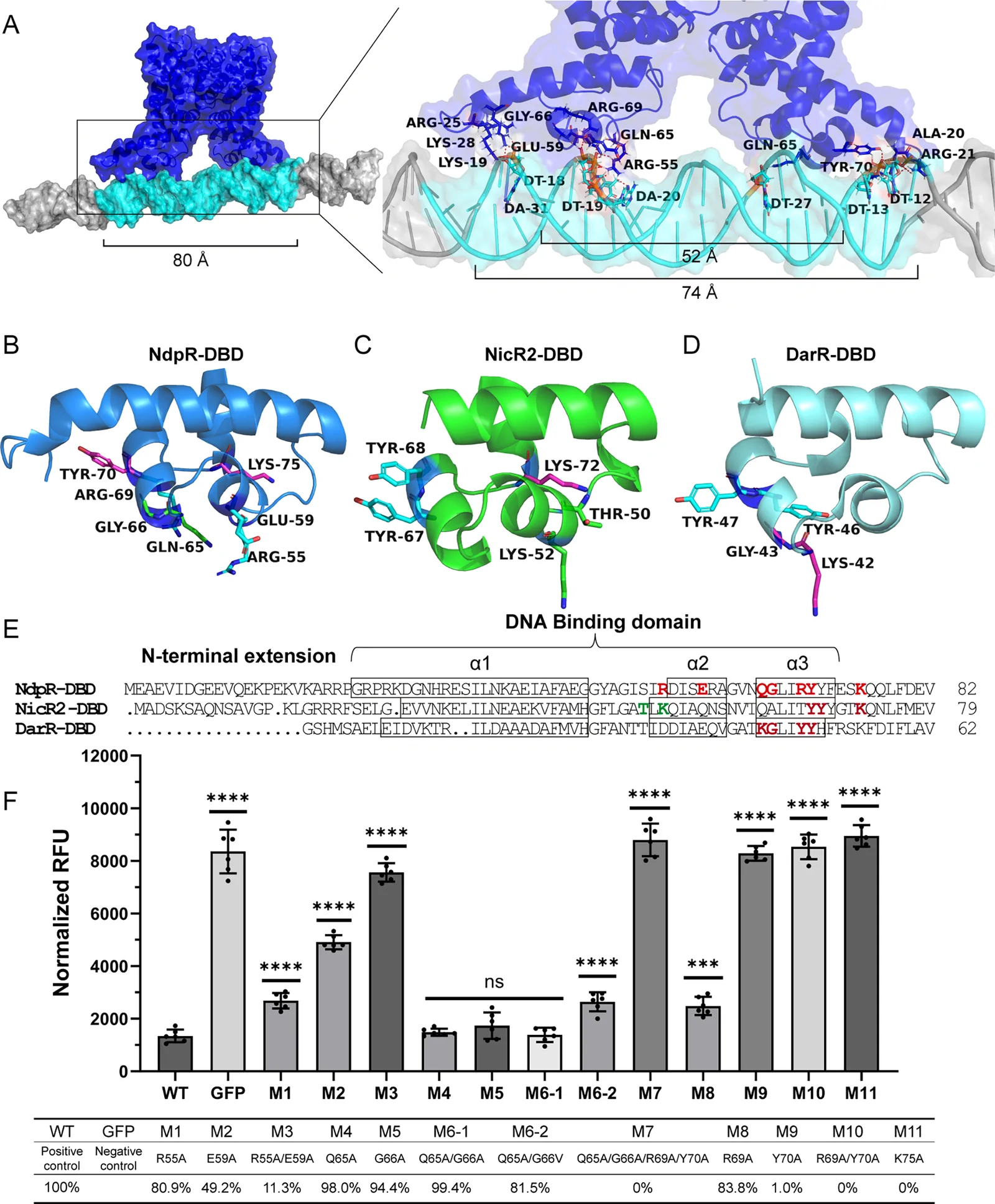
Binding mode of the NdpR homodimer to the target P*_ndpR2_* promoter motif and identification of key residues within the NdpR DBD involved in P*_ndpR2_* recognition. (**A**) Molecular docking simulation of the binding mode between the NdpR homodimer and the P*_ndpR2_* promoter. (Left) Predicted global structure of the NdpR-DNA complex. The NdpR homodimer is rendered in dark blue. The bound DNA fragment consists of the bright blue target P*_ndpR2_* motif (approximately 80 Å in length) flanked by 10-bp protective sequences on each side (shown in silver). (Right) Enlarged view of the protein-DNA interface. The homodimer covers a DNA stretch of approximately 74 Å, with a 52 Å distance between the recognition α3 helices of the two DBDs. Detailed residue-level interactions are shown, with amino acids from NdpR (dark blue) and nucleotides from the DNA (bright blue) illustrated in stick representation. Red-dashed lines represent hydrogen bond interactions. (**B through D**) Structural comparison of the DNA-binding domains. The panels show (**B**) the predicted structure of the NdpR DBD (blue), (**C**) the crystal structure of NicR2 DBD, and (**D**) the crystal structure of DarR DBD (cyan). Amino acid residues involved in DNA interactions are highlighted and color-coded based on their interaction intensities: magenta (critical), bright blue (contributing), and green (weak). (**E**) Multiple sequence alignment of the DBDs from NdpR, NicR2, and DarR. Residues experimentally verified to interact with DNA in NicR2 or DarR are indicated in dark red (strong interaction) and green (weak interaction). Potential DNA-binding residues in NdpR, inferred from the alignment and structural modeling, are highlighted in bright red. Helices α1–α3 of the HTH motif and the N-terminal extension are indicated above the alignment. (**F**) Functional impact of site-directed mutations in NdpR on the transcriptional repression of the P*_ndpR2_* promoter. The bar graph illustrates the normalized relative RFU of GFP driven by the P*_ndpR2_* promoter in the presence of wild-type NdpR or its variants (M1–M11). WT NdpR serves as a positive control for repression (activity set to 100%), while the “GFP” group represents the empty vector control (negative control). Percentages below the table indicate the residual repression activity of each mutant relative to WT. Data are presented as mean ± SD from at least three independent experiments. Statistical significance was determined by one-way ANOVA: ns, *P* > 0.05; ***P < 0.001; ****P < 0.0001.

To elucidate the molecular mechanism of DNA recognition and transcriptional repression, the structural model of the NdpR homodimer complexed with a 44-bp double-stranded DNA fragment was constructed using ZDOCK 3.0. This DNA fragment contained the 24-bp core P*_ndpR2_* operator flanked by 10-bp protective sequences at both termini, spanning an axial length of approximately 80 Å. The simulation reveals that the NdpR homodimer clamps onto the major grooves of the target DNA double helix (Fig. 4A, left).

The NdpR homodimer exhibits an exceptionally large geometric span between its DBDs. In this model, the center-to-center distance between the two α3 recognition helices (designated as the inter-HTH span) measures 52 Å. This distance significantly exceeds those reported for TetR-family homologs such as NicR2 (41.3 Å), DarR (41 Å), and PhlH (34 Å). Consequently, the NdpR homodimer exerts an axial footprint of approximately 74 Å along the DNA helix, sufficient to occupy the entire 24-bp core operator region.

An enlarged view of the interface (Fig. 4B, right) details a dense network of hydrogen bonds connecting NdpR residues to the DNA phosphate backbone and base edges. NdpR establishes specific base contacts primarily by inserting its α3 recognition helices into the major grooves. Additionally, residues within the N-terminal extension preceding helix α1 provide non-specific electrostatic interactions that further stabilize the protein-DNA complex. According to the docking model, residues potentially involved in DNA interactions included Arg55 and Glu59 on helix α2, Gln65, Gly66, Arg69, and Tyr70 on helix α3, as well as residues in the N-terminal extension. Given that NdpR-mediated repression of *ndpR2* represents the first transcriptional layer of the regulatory cascade, identifying the key residues required for NdpR recognition of P*_ndpR2_* is essential for clarifying how this regulatory hierarchy is established at the molecular level.

### Prediction and functional validation of key DNA-binding residues in NdpR

NdpR DBD (Fig. 4B) was aligned with its homologs NicR2 (Fig. 4C) and DarR (Fig. 4D) (17) to identify the conserved structural features. The amino acid residues involved in DNA interactions in NicR2 and DarR are shown as sticks in Fig. 4C and D. Structural superposition highlighted high folding similarity within the recognition helices. We prioritized residues located on helices α2 and α3 because these helices form the major DNA-recognition region of TetR-family regulators. By combining multiple sequence alignment (Fig. 4E) with our molecular docking simulations, we pinpointed seven residues as putative DNA-binding sites: Arg55 and Glu59 on helix α2; Gln65, Gly66, Arg69, and Tyr70 on helix α3; and Lys75 flanking α3. These predicted residues were then selected for single and combined alanine-substitution mutagenesis to experimentally evaluate their contribution to NdpR-mediated repression of P*_ndpR2_*.

To validate these predictions, a series of single and combinatorial alanine-substitution mutants were constructed and assessed using a P*_ndpR2_-gfp* reporter system in *E. coli* DH5α. As shown in Fig. 4F, relative to wild-type NdpR (100% repression), single mutations on helix α2, including NdpR(R55A) and NdpR(E59A), significantly diminished repression activity. Among these mutants, NdpR(E59A) retained only 49.2% of the WT activity. The NdpR(R55A-E59A) double mutant showed a further reduction in repression activity relative to the corresponding single mutants, with repression falling to 11.3%.

On helix α3, alanine substitutions at positions Gln65 and Gly66, including NdpR(Q65A), NdpR(G66A), and the NdpR(Q65A-G66A) double mutant, did not significantly affect repression activity. However, replacement of Gly66 with a bulkier and more hydrophobic residue, NdpR(G66V), reduced repression activity to 81.5%. Residues Arg69 and Tyr70 were found to be critical for NdpR function. NdpR(R69A) retained 83.8% activity, whereas NdpR(Y70A) almost completely abolished repression of the P*_ndpR2_* promoter (1.0% residual activity). Both the NdpR(R69A-Y70A) double mutant and the NdpR(Q65A-G66A-R69A-Y70A) quadruple mutant completely lost repression activity. Furthermore, NdpR(K75A) also resulted in a complete loss of regulatory function, indicating that Lys75 flanking helix α3 is critical for NdpR. This finding is consistent with previous mutational evidence for the NicR2 homolog (Fig. 4F), in which the conserved corresponding residue Lys72 was shown to be important for DNA binding (18).

### Molecular docking of NdpR with 2,5-DHP and protein-protein docking of the NdpR-NdpR2 complex

**Fig 5:**
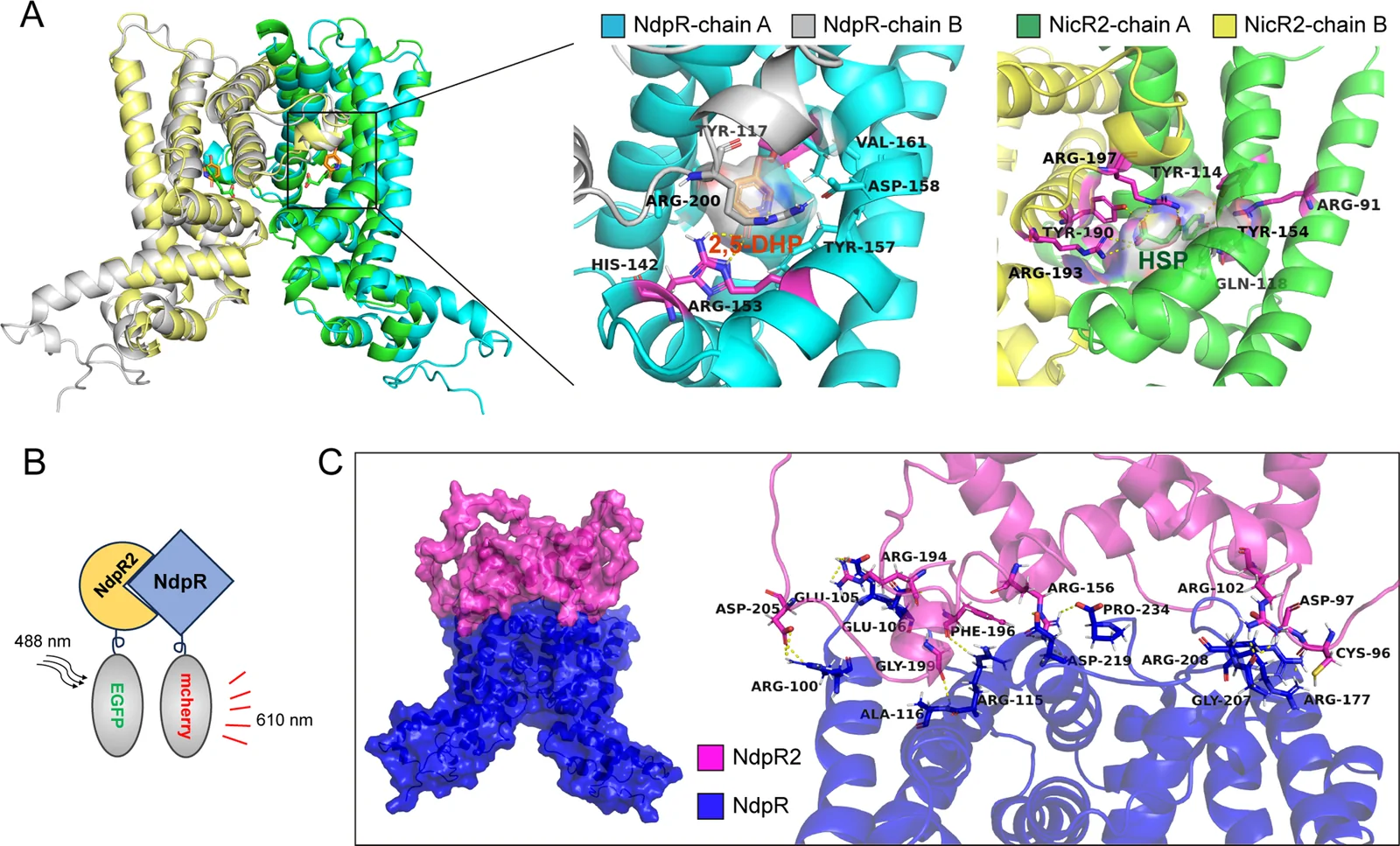
Comparative analysis of effector binding pockets of NdpR and NicR2 and the NdpR-NdpR2 interaction model. (**A**) Structural superposition of the effector-binding pockets of NdpR and NicR2. (Left) Global alignment of the NdpR homodimer (cyan and gray) and the NicR2 homodimer (green and yellow; PDB ID: 5GFL). (Middle and Right) Close-up views of the binding pockets for NdpR in complex with 2,5-DHP (orange sticks) and NicR2 in complex with HSP (green sticks). Key residues involved in ligand recognition are illustrated in stick representation. (**B**) Schematic representation of the FRET system for detecting NdpR-NdpR2 interaction. The activator NdpR2 and repressor NdpR are fused to EGFP and mCherry, respectively, to evaluate the protein-protein interactions via FRET measured at 610 nm upon excitation at 488 nm. (**C**) Molecular docking model of the NdpR-NdpR2 complex. (Left) Predicted docking orientation between the NdpR homodimer (blue surface) and the NdpR2 monomer (magenta surface). (Right) Enlarged view of the PPI interface. Interacting residues are shown in sticks colored by their respective proteins. Yellow-dashed lines indicate potential hydrogen bond interactions.

To understand the structural basis of signal sensing, the binding mode of 2,5-DHP within the NdpR homodimer was modeled. In solution, 2,5-DHP exists predominantly as its lactam tautomer, 2-pyridone-5-ol. Molecular docking using AutoDock predicted a ΔG of −5.1 kcal/mol for this ligand. The model indicates that the ligand-binding pocket is a buried internal cavity defined by helices α5, α6, α7, and α8 from one monomer, and helix α9 from the adjacent monomer (Fig. 5A). In this model, the ligand is positioned with its hydroxyl group flanked by hydrophobic residues. The predicted interactions involve Arg153, His142, and Tyr157 from one subunit and Arg200 from the other.

To validate the predicted binding pocket, we individually substituted the four predicted key residues, His142, Arg153, Tyr157, and Arg200, with alanine and examined their functional effects by EMSA in the presence of increasing concentrations of 2,5-DHP (Fig. S4). Compared with WT NdpR, NdpR(H142A), NdpR(Y157A), and NdpR(R200A) showed reduced sensitivity to 2,5-DHP, whereas NdpR(R153A) behaved similarly to WT NdpR. These results support the docking-based prediction and suggest that His142, Tyr157, and Arg200 are likely involved in 2,5-DHP-mediated modulation of NdpR DNA-binding activity.

Comparative analysis with NicR2 (which binds HSP) shows that while their effector-binding pockets occupy the same topological site, they differ in volume and shape: the NdpR pocket is 108.54 Å³, whereas the NicR2 pocket is approximately 121 Å³ (18). Despite these differences, the types of residues involved in ligand recognition, such as arginine and tyrosine, are similar between the two proteins (Fig. S5).

Having examined the predicted effector-recognition mechanism of NdpR, we next investigated the possible structural basis for its interaction with NdpR2 in the regulatory cascade. Previous FRET experiments (29) suggested physical proximity between NdpR and NdpR2, providing background support and a rationale for modeling the NdpR-NdpR2 complex (Fig. 5B). We performed *de novo* structural modeling of NdpR2 utilizing AlphaFold2. The structural accuracy was evaluated by subjecting the key DBDs to amino acid sequence alignment and structural superposition with the known crystal structures of CdpR (48) and AraT (Fig. S6). Protein-protein docking was performed using ZDOCK. The simulation yielded a complex model with a ΔG of −66.76 kcal/mol. In this model, NdpR2 and the NdpR homodimer form an extensive interface stabilized by a dense network of hydrogen bonds (Fig. 5C). Notably, the predicted NdpR2-contacting surface on NdpR is located away from the N-terminal DBD and does not overlap with the 2,5-DHP-binding pocket. An enlarged annotated view is provided in Fig. S7 to show the relative positions of the predicted protein-protein contacting surface, the 2,5-DHP-binding pocket, and the DNA-binding site.

### Construction and functional analysis of the multi-factor regulatory system

To evaluate the application potential of the NdpR-NdpR2 regulatory cascade in synthetic biology, we constructed a multi-factor control system in *Pseudomonas* sp. JY-QΔ*Nic1-2* (a transport-proficient but degradation-deficient chassis, unpublished). The system was hosted on plasmid pMTF-A/R2, which integrated P*_lac_-ndpR*, P*_ndpR2_-ndpR2*, and a *gfp* reporter module driven by either the P*_ndpR2_* or P*_ndpA_* promoter (Fig. S8A).

The results showed that the engineered system responded to different combinations of external signals (Fig. S8B). In the basal state (absence of inducers), detectable fluorescence output was observed, suggesting basal activity of the reporter promoters in the reconstructed system. JY-QΔ*Nic1-2*-A and JY-QΔ*Nic1-2*-R2 represent the P*_ndpA_-gfp* and P*_ndpR2_-gfp* reporter strains, respectively. Under different inducer treatments, the two reporter strains showed similar overall response trends, with the main difference being the expression strengths of the two promoters. Addition of the substrate nicotine significantly increased normalized RFU levels, while the subsequent addition of IPTG to the nicotine-induced state resulted in a distinct attenuation of fluorescence intensity. Conversely, the addition of 2,5-DHP alone strongly suppressed transcriptional output. Notably, the introduction of IPTG into this 2,5-DHP-inhibited state resulted in a significant recovery of fluorescence compared with the 2,5-DHP-only group (Fig. S8B).

These results indicate that the reconstructed regulatory module can respond to multiple chemical inputs in a heterologous host, although the detectable basal fluorescence suggests that further optimization will be required to minimize basal leakage before practical applications.

## DISCUSSION

Microorganisms encounter diverse environmental stimuli in rapidly changing ecosystems, making precise and sensitive gene regulatory networks crucial for their survival, adaptation, and optimal resource allocation. During the degradation of complex pollutants, substrate uptake, transport, and catabolism are typically co-regulated by multiple factors. These regulators control the expression of numerous target genes and can interact with one another to form complex intracellular regulatory networks (49–51). Elucidating these mechanisms deepens our understanding of microbial metabolic functions and provides insights into regulatory elements for future development and applications (52, 53). In this study, we reveal a cascade regulatory mechanism in *S. melonis* TY, in which NdpR and NdpR2 interact at both the transcriptional and protein levels to coordinate nicotine degradation. These findings further refine the regulatory framework of nicotine degradation in this strain by linking transcriptional repression, effector sensing, protein-protein interaction, and activator-mediated transcriptional activation. This regulatory architecture provides a mechanistic framework for understanding how nicotine-degrading bacteria balance metabolic readiness with the avoidance of unnecessary pathway expression.

Previous studies have demonstrated that transcription of nicotine-degrading genes within the *ndp* cluster is strongly induced by nicotine (35, 41). In addition, NdpR and NdpR2 were shown to coregulate the majority of the metabolism-related genes in the *ndp* cluster except *ndpC* (29). The present study further reveals that in addition to the degradation genes, the transcription of the regulatory protein-encoding genes *ndpR* and *ndpR2* is also induced by nicotine (Fig. S1). Through further investigation, we demonstrated that NdpR can directly bind to the P*_ndpR2_* promoter and negatively regulate its transcription, whereas the transcription of *ndpR* is not controlled by NdpR2. This finding establishes a cascade regulation that places NdpR upstream of the activator NdpR2. In the absence of inducing conditions, this hierarchy likely enables NdpR to maintain the nicotine metabolic module in a repressed basal state. Upon nicotine conditions, the transcriptional repression of *ndpR2* by NdpR is relieved, and increased *ndpR2* expression allows NdpR2 to function as an amplification node that activates nicotine metabolism-related promoters. This regulatory cascade enables cells to buffer or amplify environmental signals through a regulatory hub.

RT-qPCR-based transcriptional analysis further supports the functional hierarchy of this cascade system (Fig. S1). Under non-nicotine conditions, NdpR likely serves as the primary transcriptional repressor in the basal state to pre-repress the nicotine metabolic module. This interpretation is congruent with the phenotype of the TYΔ*ndpR* mutant, which responds more rapidly to nicotine in the uninduced state (28). When nicotine was utilized as the sole carbon and nitrogen source, both *ndpR* and *ndpR2* were significantly upregulated, verifying their direct involvement in the nicotine metabolic response (Fig. S1). Notably, *ndpR2* remained consistently higher than that of *ndpR* across various growth stages, likely due to the positive autoregulation of NdpR2 (29). This expression pattern suggests that the regulatory network may adjust the relative abundance of these two regulators according to the metabolic state, thereby coordinating basal repression with nicotine-induced pathway activation.

Consistent with this transcriptional hierarchy, NdpR represses the transcription of the *ndpR2* gene, while *ndpR* transcription is not governed by NdpR2. EMSA and DNA footprinting analyses further showed that NdpR2 lacks direct binding affinity for P*_ndpR_* (Fig. 2D), whereas NdpR directly binds to the P*_ndpR2_* promoter (Fig. 1C and D). Notably, this binding region shares a highly conserved recognition motif with the previously delineated NdpR-binding sites on P*_ndpA_*, P*_ndpH_*, and P*_ndpT_* (28). Together, these findings define a regulatory cascade in which NdpR controls the expression of the activator NdpR2, thereby linking basal repression with nicotine-induced transcriptional amplification.

The structural and mutational analyses further explain how NdpR achieves stringent repression of target promoters. Compared with typical TetR family members such as NicR2, DarR, and PhlH (16–18), the NdpR homodimer possesses a broader spatial arrangement of its DBDs, which supports stable occupancy of extended operator regions and steric interference with transcription initiation. Site-directed mutagenesis further corroborated the functional hierarchy of the interfacial residues within this structure (Fig. 4). Mutational analysis identified several key residues that contribute to NdpR-mediated repression, especially residues within or adjacent to the DNA-recognition helices. Among these residues, Tyr70 and Lys75 are critical for maintaining the DNA-binding activity of NdpR, whereas Arg55 and Glu59 likely stabilize the protein-DNA interface through cumulative electrostatic or hydrogen-bonding interactions. The Gly66 site is also noteworthy: alanine substitution was tolerated, but replacement with the bulkier valine impaired repression capacity, suggesting that this locus is sensitive to steric constraints within the DNA-recognition interface. Additionally, the conservation of Lys75 among diverse TetR family regulators (Fig. 3C), coupled with its critical role in NdpR (18) and NicR2, underscored the conserved role of this lysine residue in anchoring the HTH motif. The residue variations between the DBD of NdpR and its homologous proteins likely signify an evolutionary fine-tuning strategy. This permits these proteins to leverage a conserved structural scaffold while maintaining highly specific recognition of their respective promoter targets through localized residue substitutions.

The system’s perception of metabolic progression is mediated by the concentration of the intermediate 2,5-DHP. In this cascade, 2,5-DHP acts as both an inducer for NdpR and a negative effector for NdpR2. *In vitro* EMSA experiments revealed that varying concentrations of the inducer 2,5-DHP are required to compel the complete dissociation of NdpR from its diverse target genes. Specifically, P*_ndpA_* and P*_ndpH_* necessitate 3 mM, while P*_ndpT_* (28) and P*_ndpR2_* require merely 1 mM (Fig. 2B). Similarly, the concentrations required for 2,5-DHP, functioning as a negative effector, to disrupt the binding of NdpR2 to its target genes also vary. However, all these thresholds remain below 1 mM (29). This concentration gradient response indicates that the two regulators do not execute a binary on/off switch; rather, they may rely on effector-regulator interactions to perceive minute concentration fluctuations of 2,5-DHP, achieving dynamic transcriptional regulation across distinct target genes.

The specific effector recognition by NdpR, alongside its homolog NicR2 (18), manifests a paradigm of positional conservation coupled with morphological specialization, empowering these homologous proteins to accurately discriminate structurally analogous metabolic intermediates within the nicotine degradation pathway. This implies that while preserving the core sensing capacity for nicotine-derived intermediates, they precisely accommodate the target-specific evolution of their unique genomic islands and metabolic routes.

Beyond transcriptional control, the direct physical association between NdpR and NdpR2 constitutes an additional regulatory layer. In our model, the “double-lock” mechanism is most prominent in the absence of nicotine. At the transcriptional level, NdpR represses *ndpR2* expression by binding to the P*_ndpR2_* promoter. At the protein-interaction level, NdpR physically contacts NdpR2, and the predicted interface (Fig. 5C) involves the DBD of NdpR2 (Fig. S6). This suggests that direct physical contact with NdpR may interfere with NdpR2 binding to promoter DNA, thereby reducing the transcriptional activation capacity of NdpR2. Notably, the predicted NdpR2-contacting surface on NdpR does not overlap with either the DNA-binding domain or the 2,5-DHP-binding pocket, suggesting that NdpR may interact with NdpR2 through a spatially distinct surface. Whether this interaction induces conformational changes in NdpR or NdpR2 remains unknown and requires further investigation. Nevertheless, this architecture, integrating transcriptional repression of activator synthesis with protein-level restriction of the synthesized activator, resembles the ExsA-ExsD anti-activator system in *P. aeruginosa* (20, 21) and may provide a multi-layered logic for orchestrating metabolic transitions.

**Fig 6:**
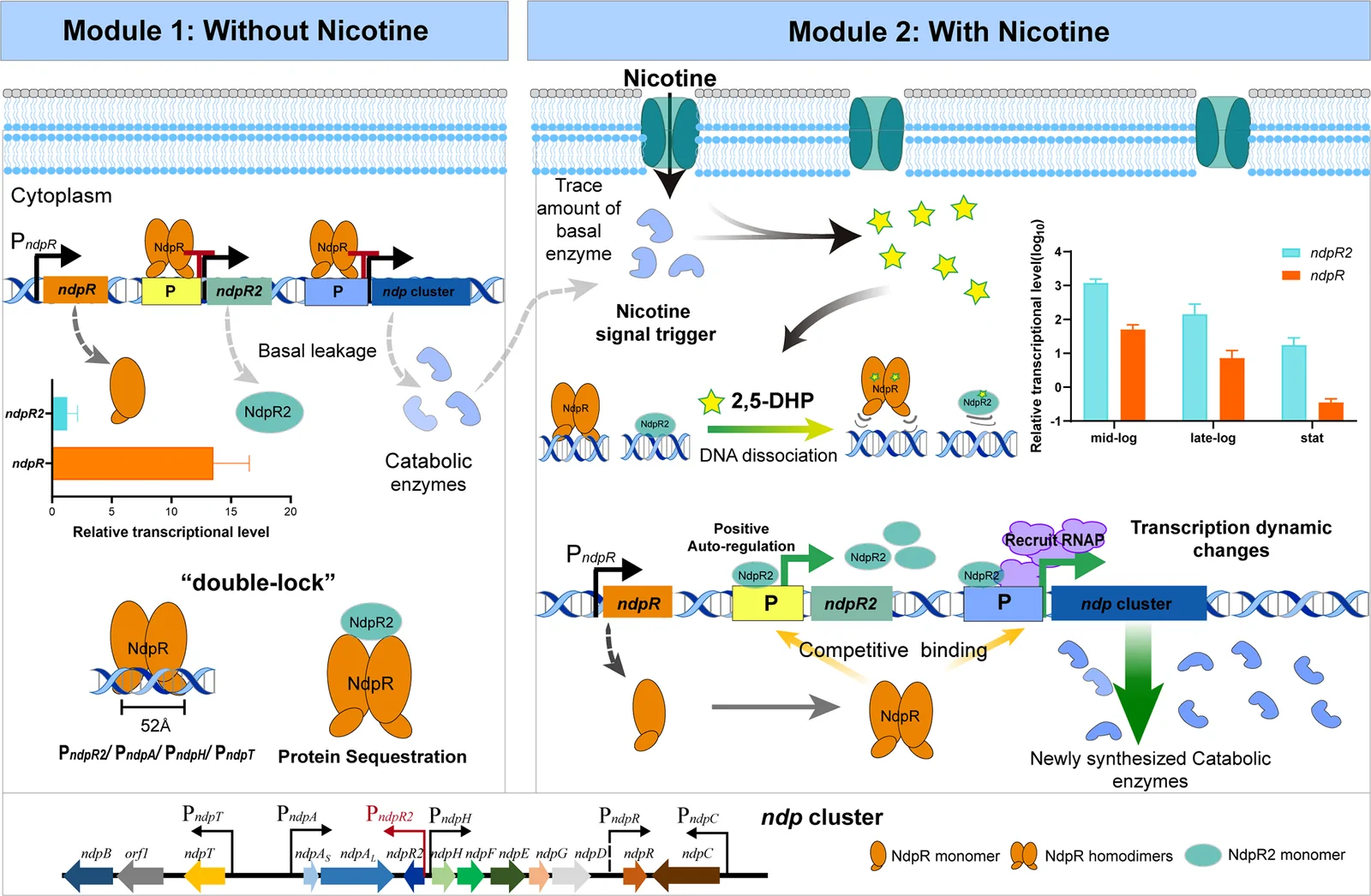
Schematic diagram of the cascade regulatory mechanism for nicotine metabolism mediated by NdpR and NdpR2 in strain TY. The left panel shows the state in the absence of nicotine, while the right panel illustrates the nicotine signal response and the dynamic cascade regulatory mode.

Based on these findings, we propose a cascade regulatory model for NdpR-NdpR2-mediated nicotine degradation (Fig. 6). In the absence of nicotine, NdpR maintains the system at a low basal expression level by repressing *ndpR2* transcription and interacting with the basal leakage of NdpR2 activity through protein-protein interaction, forming a stringent repression. When nicotine appears in the environment, it is taken into the cell by a constitutively synthesized permease (54). Trace amounts of basal degradation enzymes convert nicotine into the metabolic intermediate 2,5-DHP. Acting as a pivotal signaling molecule, 2,5-DHP relieves the transcriptional repression of NdpR on degradation enzyme genes and the activator-encoding gene *ndpR2* (28, 29), thereby allowing NdpR2 accumulation and activation of nicotine metabolism-related promoters by binding to metabolic gene promoters and efficiently recruiting RNA polymerase (7). Throughout the subsequent metabolic cascade, the dynamically fluctuating 2,5-DHP concentration continuously modulates the activities of NdpR and NdpR2, directly impacting their transcriptional regulatory efficacy. Meanwhile, the positive autoregulation of NdpR2, the transcriptional repression of *ndpR2* by NdpR, and the protein-protein interaction between the two regulators systematically recalibrate their binding stoichiometry at target promoters. This multifaceted balance is proposed to enable the metabolic pathway to elicit a precise response to substrate fluctuations, thereby circumventing metabolic leakage while guaranteeing highly efficient degradation.

Recent studies have shown that biodegradation-associated pathways and regulatory modules can be rewired for biotransformation, pathway balancing, and metabolic engineering. Examples include engineered co-culture systems coupling 2,4-dinitrotoluene (2,4-DNT) degradation with polyhydroxyalkanoate (PHA) biosynthesis (55) and the repurposing of BenR/P*_ben_* and CatR/P*_cat_* modules in engineered *P. putida* for balancing fluorinated aromatic compound production (56). To evaluate the translational potential of the NdpR-NdpR2 cascade system in synthetic biology, the pMTF-A/R2 multi-factor system was constructed in a heterologous host. (Fig. S8A) The reconstructed system responded to different combinations of nicotine, 2,5-DHP, and IPTG (Fig. S8B), and these response patterns were highly consistent with the NdpR-NdpR2 cascade regulatory mechanism resolved in this study. By harnessing the regulatory proteins NdpR and NdpR2, this system generated tunable transcriptional outputs and exhibited bidirectional regulation of the target gene expression state, supporting the functional modularity of the NdpR-NdpR2 cascade. These findings suggest that this regulatory framework may serve as a valuable tool for developing programmable multi-input gene expression systems in synthetic biology and metabolic engineering, particularly in non-model bacterial chassis. Nevertheless, the detectable basal fluorescence observed in the reconstructed system indicates that further optimization will be required before practical application, including reducing promoter leakage, tuning regulator dosage, and refining circuit architecture.

### Conclusion

In summary, this study elucidates the cascade mechanism by which NdpR and NdpR2 regulate nicotine catabolism in the *S. melonis* TY strain. We demonstrate that the upstream regulator protein NdpR directly negatively regulates the expression of the downstream activator NdpR2, while NdpR2 had no effect on the expression of *ndpR*. The metabolic intermediate 2,5-DHP functions as an effector molecule that relieves NdpR-mediated transcriptional repression. Combined with structural modeling and molecular docking, we analyzed how NdpR represses target promoters and binds the effector 2,5-DHP, and mutational analysis further validated the key amino acid residues involved in these processes. Molecular docking also suggested that the interaction between NdpR and NdpR2 may affect the activation capacity of NdpR2. Based on these findings, we proposed a double-lock regulatory mechanism that operates synergistically at both the transcriptional and protein levels. This precise mechanism ensures that cells can sensitively respond to environmental signals relying on basal metabolism and efficiently initiate target gene expression upon signal confirmation, thereby achieving a balance between energy economy and efficient substrate degradation. Finally, based on this cascade system and utilizing a *gfp* reporter gene, we successfully constructed a dynamically tunable multi-factor gene regulatory circuit in the heterologous host *Pseudomonas* sp. JY-Q. This study not only deepens our understanding of microbial environmental adaptation mechanisms but also expands the toolbox of programmable regulatory elements for synthetic biology. In future applications, this regulatory cascade could be further optimized by reducing basal leakage, tuning regulator dosage, and refining promoter strength, thereby enabling its use in programmable multi-input gene expression systems, metabolic pathway control, and engineered degradation or biosynthesis platforms across various types of bacterial chassis.

## Data Availability

The experimental data sets generated in this study are provided in the article and supplemental material or are available from the corresponding author upon reasonable request for non-commercial scientific purposes, including verification, reproduction, or reanalysis. No new nucleotide or protein sequences, software, algorithms, or custom code were generated. The genome and associated sequence data of *Sphingomonas melonis* TY are publicly available in NCBI GenBank under accession number CP017578.1 (Ref-seq accession NZ_CP017578.1).

## SUPPLEMENTAL MATERIAL

Supplemental material aem.01130-26-s0001.docx Supplemental methods, Fig. S1 to S8, and Tables S1 to S3.

ASM does not own the copyrights to Supplemental Material that may be linked to, or accessed through, an article. The authors have granted ASM a non-exclusive, world-wide license to publish the Supplemental Material files. Please contact the corresponding author directly for reuse.
