# Supplementary material for: Regulatory cascade of NdpR and NdpR2 in controlling nicotine degradation operons in Sphingomonas melonis TY
Source: Appl Environ Microbiol. 2026 Aug 12;92(9):e01130-26. doi: 10.1128/aem.01130-26 (PMC13599538; doi:10.1128/aem.01130-26)
Supplement: Supplementary file 1 [file aem.01130-26-s0001.docx]

**Regulatory Cascade of NdpR and NdpR2 in Controlling Nicotine Degradation Operons in *Sphingomonas melonis* TY**

Qi Tang,^a^ Ziliang He,^a^ ShihanWang,^a^ Yiming Cheng,^a^ Haixia Wang,^a^ Weihong Zhong,^a^

^a^ College of Biotechnology and Bioengineering, Zhejiang University of Technology, Hangzhou, China

# Supplementary materials

# Methods

## RT-qPCR analysis.

Wild-type TY grown with nicotine or glucose as the sole carbon source at different growth phases was sampled for RT-qPCR analysis of the *ndpR* and *ndpR2* genes. Samples were collected at defined growth phases, including mid-log, late-log, and stationary phases, as determined based on the growth curve. The late-log phase was defined as the stage immediately before entry into the stationary phase, while stationary-phase samples were collected 12 h after the culture reached the growth plateau. All samples were rapidly harvested and used for subsequent RNA extraction. Additionally, wild-type TY and the TYΔ*ndpR* mutant grown with nicotine were sampled at the mid-log phase for *ndpR2* expression analysis. Total RNA was extracted using RNAiso Plus (Takara, Japan) following the manufacturer’s protocol. cDNA synthesis was performed using random hexamer primers and the PrimeScript™ RT reagent Kit with gDNA Eraser (Perfect Real Time) (Takara, Japan), and the cDNA was stored at -20 °C until use.

RT-qPCR was conducted on a real-time PCR system with a SYBR Green-based detection method. Reactions were prepared in 96-well plates using ChamQ Universal SYBR qPCR Master Mix (Vazyme, Nanjing, China) in a final volume of 20 μL. Each reaction contained molecular biology-grade water, 1 × SYBR qPCR Master Mix, 0.2 μM of forward and reverse primers, and 1-10 ng of cDNA template. Amplifications were carried out on a CFX Connect™ Real-Time System (Bio-Rad, Hercules, Germany) under the following conditions: 95 °C for 30 s, followed by 40 cycles of 95 °C for 10 s and 60 °C for 30 s, with a final melting curve analysis (65-95 °C, 0.5 °C increments) to confirm specificity.

Relative gene expression was calculated using the 2^^-ΔΔCt^ method, with normalization to the reference gene 16S rRNA. The primers used are listed in Table S2. No-template controls were included to check for contamination and served as negative controls. All experiments included three biological replicates and four technical replicates per sample.

## Reporter plasmid construction and β-galactosidase assay.

A 400-bp fragment containing the TSSs of the tested operons (spanning from 300 bp upstream to 100 bp downstream of the start codon) was amplified from *S. melonis* TY genomic DNA using specific primer pairs (Table S2) to obtain P*_ndpR2_* and P*_ndpR_*. The PCR products were fused with *Eco*R Ⅰ/*Kpn* Ⅰ digested pHGR18670 using the In-Fusion HD cloning kit (TaKaRa, Dalian, China) to generate the corresponding integrative reporter plasmids pHGR18670-P*_ndpR_* and pHGR18670-P*_ndpR2_*, which carry the P*_ndpR_*-*lacZ* and P*_ndpR2_*-*lacZ* transcriptional fusions, respectively. These constructed reporter plasmids were conjugated into wild-type TY, TYΔ*ndpR*, and TYΔ*ndpR2*.

β-galactosidase assays were conducted as described previously(1). β-Galactosidase assays were performed in wild-type TY, TYΔ*ndpR*, and TYΔ*ndpR2* grown in NaCl-free LB medium to evaluate promoter activity. Specifically, TYΔ*ndpR*-pHGR18670-P*_ndpR2_*-lacZ was compared with the control strain TY-pHGR18670-P*_ndpR2_*-*lacZ*, while TYΔ*ndpR2*-pHGR18670-P*_ndpR_*-*lacZ* was compared with TY-pHGR18670-P*_ndpR_*-*lacZ*. β-Galactosidase activity was measured using 4 mg/mL o-nitrophenyl-β-D-galactopyranoside as the substrate. Absorbance at 420 nm was recorded and normalized to OD_600_, and enzyme activity was calculated as OD_420_ × 1,000 ÷ (OD_600_ × *v* × *t*), where *v* is the sample volume and *t* is the reaction time. One Miller unit is defined as the amount of enzyme required to produce 1 μmol of o-nitrophenol per minute.

## EMSA

NdpR and NdpR2 were purified as described previously. DNA fragments for EMSA were amplified from *S. melonis* TY genomic DNA using primers listed in Table S2 and purified using a DNA fragment purification kit (Tiangen Biotech Co., Ltd., Beijing, China). The promoter region F0 was defined as a 400-bp fragment containing the TSS of the tested operons, spanning from 300 bp upstream to 100 bp downstream of the start codon. DNA regions showing protein-binding activity were subdivided into overlapping segments to progressively narrow down the binding region and guide the design of subcloned fragments. For binding reactions, approximately 10 nM of the F0 fragment or 20 nM of other subclone fragments was mixed with 200 nM purified NdpR in binding buffer (50 mM sodium phosphate [pH 7.0], 300 mM NaCl, 2 mM EDTA, and 5% glycerol [v/v]) to a final volume of 25 μL. For NdpR2, a different binding buffer was used (10 mM Tris-HCl [pH 7.5], 80 mM NaCl, 1 mM EDTA, 20 mM β-mercaptoethanol, and 4% glycerol [v/v]). Binding reaction mixtures were incubated at 30°C for 25 minutes before being subjected to electrophoresis on a non-denaturing polyacrylamide gel(2, 3).

Reactions containing the F0 fragment were separated using 8% native polyacrylamide gel electrophoresis (PAGE) in 1×TAE buffer, while a 10% gel was used for other subclone fragments. Nucleic acid-protein complexes exhibited slower migration through the gel, resulting in shifted bands indicative of binding. To assess potential effectors, 2 mM nicotine or other intermediates (6HN, 6HPON, HSP, 2,5-DHP, maleamic acid, maleic acid, or fumaric acid) were pre-incubated with NdpR at 30 °C for 15 minutes. DNA fragments were then added, and the mixture was incubated for an additional 25 minutes before electrophoresis. After electrophoresis, the gel was carefully removed from the cassette and incubated with 50 mL of 1× TAE containing 0.01% Gelstain Red (US Everbright® Inc.; Suzhou Yuheng Biotechnology Co., Ltd., Xiancheng, China) in a horizontal decolorization shaker at 50 rpm for 20 minutes at room temperature. The gel was subsequently washed twice with 150 mL of MilliQ water and visualized using standard 300 nm UV transillumination equipment.

## DNase I footprinting assay

The DNase I footprinting assay used in this study was according to previously described method(4). To prepare 6-carboxyfluorescein (FAM) labeled probe, the *ndpR2* promoter region was amplified from the pClone-NdpR2 plasmid using primers FAM-M13F and M13R. DNase I footprinting analysis was performed as previously described(5). Briefly, the labeled probe was incubated with NdpR protein (0.5, 2, 5 μg) in EMSA binding buffer, with a total volume of 40 μL. Following incubation at 25 °C for 30 min, 10 μL of a solution containing approximately 0.015 U of DNase I (Promega, Madison, USA) and 100 nmol of freshly prepared CaCl₂ was added, and the mixture was further incubated at 37 °C for 1 min. The reaction was terminated by adding 140 μL of DNase I stop solution (200 mM unbuffered sodium acetate, 30 mM EDTA, and 0.15% SDS). DNA samples were subsequently extracted with phenol-chloroform and precipitated with ethanol. The resulting pellets were resuspended in 30 μL of ultrapure water. DNA ladder preparation, electrophoresis, and data analysis were performed as previously described(5), except that the GeneScan-LIZ600 size standard (Applied Biosystems, Foster City, USA) was used.

# Figure legends

## **Fig. S1** Expression analysis of *ndpR* and *ndpR2* in wild-type TY under different growth phases and carbon sources.

Quantitative reverse transcription PCR was performed to analyze the expression of ndpR and ndpR2 in wild-type TY during the mid-logarithmic (mid-log), late-logarithmic (late-log), and stationary phases (stat) when grown with nicotine or glucose as the sole carbon source. The 16S rRNA gene was used as an internal reference for normalization. Expression levels were calculated using the 2^^-ΔΔCt^ method, based on data from three biological replicates. Error bars represent the standard error of the mean. “n.d.” means not detected (below the limit of detection; Ct > 40).

## **Fig. S2** Promoter activity of P*_ndpR2_* was evaluated using β-galactosidase assays.

Specific β-galactosidase activity measured from promoter P*_ndpR2_* in TYΔ*ndpR* (pHGL::*ndpR*) and TYΔ*ndpR*(pHGL) strains.

## Fig. S3 Structural models and validation of the NdpR monomer and homodimer.

(A) Predicted 3D structure of the NdpR monomer generated using AlphaFold2. The model showed a mean pLDDT score of 84.6 and a pTM score of 0.783. (B) Ramachandran plot analysis of the predicted NdpR monomer model. A total of 94.4% of residues were located in favored regions. (C) Predicted 3D structure of the NdpR homodimer generated using AlphaFold2-multimer. The model showed a mean pLDDT score of 82.6, a pTM score of 0.804, and an ipTM score of 0.799, indicating reasonable confidence in the overall fold and predicted dimer interface.

## Fig. S4 EMSA analysis of the effect of predicted 2,5-DHP binding pocket mutations of NdpR.

The promoter sequence DNA (P*_ndpR2_* F0 fragment, 20 nM) was incubated with each purified NdpR point mutant (200 nM) in the presence of increasing concentrations of 2,5-DHP. “CK” represents the control group without NdpR. (A) NdpR(H142A) and NdpR(R153A); (B) NdpR(Y157A) and NdpR(R200A).

## Fig. S5 Two-dimensional representation of the effector-binding interactions in NicR2 and NdpR.

(A) 2D schematic of the NicR2 effector-binding pocket in complex with HSP. Specific residues involved in ligand recognition, including Arg91, Gln118, and Arg197, are indicated. (B) 2D schematic of the NdpR effector-binding pocket in complex with 2,5-DHP. Key residues stabilizing the ligand, such as Arg153, His142, and Arg200, are highlighted. In both panels, green dashed lines represent hydrogen bond interactions (with bond distances labeled in Å), while red semi-circles with radiating spokes denote residues participating in hydrophobic or van der Waals contacts.

## Fig. S6 Modeling and comparative analysis of the NdpR2 monomer.

(A) Predicted 3D structure. The monomer is shown in rainbow-colored ribbon representation. The model showed a pTM score of 0.524. (B) Ramachandran plot. Stereochemical validation shows 93.5% of residues in the favored regions. (C) Structural superposition. The NdpR2 DNA-binding domain (DBD) is aligned with homologs AraT (PDB ID: 6SWI) and CdpR (PDB ID: 5CHH). (D) Sequence alignment. MSA of the NdpR2 DBD with secondary structure elements (α1- α7) indicated above.

## Fig. S7. Enlarged annotated view of the protein-protein docking model of the NdpR-NdpR2 complex.

The NdpR homodimer is shown in blue, and the NdpR2 monomer is shown in magenta. The predicted protein-protein contacting surface, the 2,5-DHP-binding pocket, and the DNA-binding site of NdpR are indicated by black, green, and blue ellipses, respectively. This enlarged view shows that the predicted NdpR2-contacting surface is spatially separated from the DNA-binding site and does not overlap with the predicted 2,5-DHP-binding pocket.

## Fig. S8 Construction and functional validation of the multi-factor regulatory system.

(A) Schematic representation of the multi-factor regulatory plasmid pMTF-A/R2. The reporter gene *gfp* is driven by either the P*_ndpA_* or P*_ndpR2_* promoter, as highlighted in red. The plasmid contains the IPTG-inducible P*_lac_*-*ndpR* module and *ndpR2*, enabling reconstruction of the NdpR-NdpR2 regulatory cascade for multi-input logic control.

(B) Transcriptional output of the regulatory system under different induction conditions. JY-QΔ*Nic1-2*-A and JY-QΔ*Nic1-2*-R2 represent the P*_ndpA_*-*gfp* and P*_ndpR2_*-*gfp* reporter strains, respectively, and are shown as black and gray bars. The presence (+) or absence (-) of nicotine, 2,5-DHP, and IPTG is indicated below the x-axis. Data represent the mean ± SD from three independent biological replicates. Statistical significance was evaluated using one-way ANOVA: ns, not significant (P > 0.05); ***, P < 0.001; ****, P < 0.0001.

**Induction response analysis of the multi-factor regulatory system**

To evaluate the translational potential of this cascade system in synthetic biology, we constructed the pMTF-A/R2 multi-factor system in a heterologous host in vivo, integrating a GFP reporter system to authenticate the dynamic response mechanism of the aforementioned cascade (Fig. S7A). In this system, the expression state of the target gene is co-determined by NdpR2 and NdpR. In the basal state devoid of exogenous inducers, the P*_lac_* promoter is partially repressed by LacI, resulting in the attenuated expression of its driven NdpR; concurrently, driven by the P*_ndpR2_* promoter, NdpR2 is constitutively expressed and exerts an activation effect on the P*_ndpR2_* or P*_ndpA_* promoter, sustaining a basal expression level.

When solely nicotine is supplemented in the basal state, JY-QΔ*Nic1-2* metabolizes a small amount of nicotine into trace amounts of 2,5-DHP, which subsequently binds to NdpR to relieve the repression, thereby activating the expression of *ndpR2*, exerting transcriptional activation on the reporter gene. When IPTG is subsequently introduced, NdpR expression is induced. Subsequently, NdpR can execute the downward attenuation of reporter gene expression through three synergistic mechanisms: first, by directly binding to the P*_ndpR2_* or P*_ndpA_* promoter to repress the transcription of *gfp*; second, by binding to the P*_ndpR2_* promoter to inhibit the transcriptional synthesis of the activator NdpR2; and third, via direct protein-protein interaction, rendering NdpR2 incapable of DNA binding. These three pathways operate in concert to successfully reverse the nicotine-mediated expression activation. Confronted with the mutual effector 2,5-DHP, the system exhibits a more sophisticated regulatory logic. When 2,5-DHP is added independently, it functions as a negative effector for NdpR2, abrogating its transcriptional activation capacity and repressing target gene expression. However, upon the subsequent addition of IPTG to induce NdpR expression, two aspects of regulation occur within the system: on one hand, the newly synthesized NdpR protein binds with free 2,5-DHP, consuming the 2,5-DHP in the system and weakening its negative inhibition on NdpR2, thereby allowing the activation function of NdpR2 to recover; on the other hand, NdpR bound with 2,5-DHP undergoes a conformational change, preventing it from occupying the P*_ndpR2_* or P*_ndpA_* promoter, thus relieving the direct repression of NdpR on the promoters. Therefore, compared to the experimental group where only 2,5-DHP is added, the introduction of IPTG can partially restore target gene expression through the molecular consumption effect and conformational regulation between the protein and the effector.

## Table S1 strains and plasmids used in this study

| Strain or plasmid | Relevant characteristics | Reference or source | |
| --- | --- | --- | --- |
| Strains |  |  | |
| *Escherichia coli* DH5α | *supE44lacU169*(80dlacZΔM15)*hsdR17 recA1 endA1 gyrA96*Δ*thi relA1* | (6) | |
| *Sphingomonas melonis* TY | Wild type, nicotine-degrading strain, G^-^, Amp^r^, Kan^s^, Tc^s^, Cam^s^ | This study | |
| JY-QΔ*Nic1-2* | Wild type *Pseudomonas* sp. JY-Q mutant *Nic1-2* cluster | unpublished | |
| JY-QΔ*Nic1-2*-R2 | JY-QΔ*Nic1-2* transformed with pMTF-R2 ,Gm^r^ | This study | |
| JY-QΔ*Nic1-2*-A | JY-QΔ*Nic1-2* transformed with pMTF-A ,Gm^r^ | This study | |
| TYΔ*ndpR* | *Sphingomonas melonis* TY mutant with *ndpR* gene replaced by tetracycline resistance gene, Amp^r^, Tc^r^ | This study | |
| TYΔ*ndpR2* | *Sphingomonas melonis* TY mutant with *ndpR2* gene replaced by kanamycin resistance gene, Amp^r^, Kan^r^ | This study | |
| BL21(DE3)-pG-Tf2  pET28a-*ndpR* | BL21(DE3) transformed with pET-28a *ndpR*, Kan^r^ and pG-Tf2, Cam^r^ | | (4) |
| M1 | *Escherichia coli* DH5α transformed with pUC19-*ndpR*(R55A)-P*_ndpR2_*-*gfp*, Amp^r^ | | This study |
| M2 | *Escherichia coli* DH5α transformed with pUC19-*ndpR*(E59A)-P*_ndpR2_*-*gfp*, Amp^r^ | | This study |
| M3 | *Escherichia coli* DH5α transformed with pUC19-*ndpR*(R55A/E59A)-P*_ndpR2_*-*gfp*, Amp^r^ | | This study |
| M4 | *Escherichia coli* DH5α transformed with pUC19-*ndpR*(Q65A)-P*_ndpR2_*-*gfp*, Amp^r^ | | This study |
| M5 | *Escherichia coli* DH5α transformed with pUC19-*ndpR*(G66A)-P*_ndpR2_*-*gfp*, Amp^r^ | | This study |
| M6-1 | *Escherichia coli* DH5α transformed with pUC19-*ndpR*(Q55A/G66A)-P*_ndpR2_*-*gfp*, Amp^r^ | | This study |
| M6-2 | *Escherichia coli* DH5α transformed with pUC19-*ndpR*(Q55A/G66V)-P*_ndpR2_*-*gfp*, Amp^r^ | | This study |
| M7 | *Escherichia coli* DH5α transformed with pUC19-*ndpR*(Q65A/G66A/R69A/Y70A)-P*_ndpR2_*-*gfp*, Amp^r^ | | This study |
| M8 | *Escherichia coli* DH5α transformed with pUC19-*ndpR*(R69A)-P*_ndpR2_*-*gfp*, Amp^r^ | | This study |
| M9 | *Escherichia coli* DH5α transformed with pUC19-*ndpR*(Y70A)-P*_ndpR2_*-*gfp*, Amp^r^ | | This study |
| M10 | *Escherichia coli* DH5α transformed with pUC19-*ndpR*(R69A/Y70A)-P*_ndpR2_*-*gfp*, Amp^r^ | | This study |
| M11 | *Escherichia coli* DH5α transformed with pUC19-*ndpR*(K75A)-P*_ndpR2_*-*gfp*, Amp^r^ | | This study |
| BL21(DE3)-pG-Tf2-pET28a *ndpR*(H142A) | BL21(DE3) transformed with pET-28a *ndpR*(H142A), Kan^r^ and pG-Tf2, Cam^r^ | | This study |
| BL21(DE3)-pG-Tf2-pET28a -*ndpR*(R153A) | BL21(DE3) transformed with pET-28a *ndpR*(R153A), Kan^r^ and pG-Tf2, Cam^r^ | | This study |
| BL21(DE3)-pG-Tf2-pET28a *ndpR*(Y157A) | BL21(DE3) transformed with pET-28a *ndpR*(Y157A), Kan^r^ and pG-Tf2, Cam^r^ | | This study |
| BL21(DE3)-pG-Tf2-pET28a *ndpR*(R200A) | BL21(DE3) transformed with pET-28a *ndpR*(R200A), Kan^r^ and pG-Tf2, Cam^r^ | | This study |
| Plasmids |  | |  |
| pHGR18670 | Reconstruct integrative vector pHGR01 with a fragment homologous to strain TY, Kan^r^ | |  |
| pHGR18670Chlo | Plasmid constructed by replacing the kanamycin resistance gene in pHGR18670 with chloromycetin resistance gene, Cam^r^ | | This study |
| pHGR18670Chlo-P*_ndpR2_* | Reporter vector with the promoter region of *ndpR2* inserted into the *Eco*R I-Kpn I restriction site of pHGR18670Chlo, which was in the 5-terminal of the *lacZ* gene | | This study |
| pHGR18670Chlo-P*_ndpR_* | Reporter vector with the promoter region of *ndpR* inserted into the *Eco*R I-Kpn I restriction site of pHGR18670Chlo, which was in the 5-terminal of the *lacZ* gene | | This study |
| pUC19-*gfp* | Transcriptional regulator screening plasmid, Amp^r^, constructed according to previous research by Cheng et al(7), with two multiple cloning sites MCS1 and MCS2 | | (3) |
| pUC19-P*_ndpR2_*-*gfp* | pUC19-gfp with *ndpR2* promoter inserting in MCS2 | | This study |
| pUC19-*ndpR*-P*_ndpR2_*-*gfp* | pUC19-P*_ndpR2_*-*gfp* with *ndpR* gene inserting in MCS1 | | This study |
| pUC19-*ndpR*(R55A)-P*_ndpR2_*-*gfp* | NdpR R55A mutation was introduced into the pUC19-*ndpR*-*gfp* plasmid | | This study |
| pUC19-*ndpR*(E59A)-P*_ndpR2_*-*gfp* | NdpR E59A mutation was introduced into the pUC19-*ndpR*-*gfp* plasmid | | This study |
| pUC19-*ndpR*(R55A-E59A)-P*_ndpR2_*-*gfp* | NdpR R55A/E59A double mutation was introduced into the pUC19-*ndpR*-*gfp* plasmid | | This study |
| pUC19-*ndpR*(Q65A)-P*_ndpR2_*-*gfp* | NdpR Q65A mutation was introduced into the pUC19-*ndpR*-*gfp* plasmid | | This study |
| pUC19-*ndpR*(G66A)-P*_ndpR2_*-*gfp* | NdpR G66A mutation was introduced into the pUC19-*ndpR*-*gfp* plasmid | | This study |
| pUC19-*ndpR*(Q65A-G66A)-P*_ndpR2_*-*gfp* | NdpR Q65A/G66A double mutation was introduced into the pUC19-*ndpR*-*gfp* plasmid | | This study |
| pUC19-*ndpR*(Q65A-G66V)-P*_ndpR2_*-*gfp* | NdpR Q65A/G66V double mutation was introduced into the pUC19-*ndpR*-*gfp* plasmid | | This study |
| pUC19-*ndpR* (Q65A-G66A-R69A-Y70A)-P*_ndpR2_*-*gfp* | NdpR Q65A/G66A/R69A/Y70A quadruple mutation was introduced into the pUC19-*ndpR*-*gfp* plasmid | | This study |
| pUC19-*ndpR*(R69A)-P*_ndpR2_*-*gfp* | NdpR R69A mutation was introduced into the pUC19-*ndpR*-*gfp* plasmid | | This study |
| pUC19-*ndpR*(Y70A)-P*_ndpR2_*-*gfp* | NdpR Y70A mutation was introduced into the pUC19-*ndpR*-*gfp* plasmid | | This study |
| pUC19-*ndpR*(R69A-Y70A)-P*_ndpR2_*-*gfp* | NdpR R69A/Y70A double mutation was introduced into the pUC19-*ndpR*-*gfp* plasmid | | This study |
| pUC19-*ndpR*(Y75A)-P*_ndpR2_*-*gfp* | NdpR Y75A mutation was introduced into the pUC19-*ndpR*-*gfp* plasmid | | This study |
| pMMB67EH | broad-host-range expression vector，integrative lacZ reporter vector，Amp^r^ | | NovoPro |
| pMGm | Plasmid constructed by replacing the ampicillin resistance gene in pMMB67EH with gentamicin resistance gene, Gm^r^ | | This study |
| pMT | pMGm with P_lacI-_*lacI* and P*_lac_*-*ndpR* | | This study |
| pMTF | pMT with *gfp* and P*_ndpR2_*-*ndpR2*-terminator | | This study |
| pMTF-A | Insert P*_ndpA_* upstream of *gfp* in pMTF | | This study |
| pMTF-R2 | Insert P*_ndpR2_* upstream of *gfp* in pMTF | | This study |
| pET-28a *ndpR*(H142A) | NdpR H142A mutation was introduced into the pET28a-*ndpR* plasmid | | This study |
| pET-28a *ndpR*(R153A) | NdpR R153A mutation was introduced into the pET28a-*ndpR* plasmid | | This study |
| pET-28a *ndpR*(Y157A) | NdpR Y157A mutation was introduced into the pET28a-*ndpR* plasmid | | This study |
| pET-28a *ndpR*(R200A) | NdpR R200A mutation was introduced into the pET28a-*ndpR* plasmid | | This study |

## Table S2 Primers used in this study

| Primer | Sequence (5’-3’) | Purpose |
| --- | --- | --- |
| 16S-F | tgacgctgaggtgcgaaagc | To amplify the 16S rRNA of strain TY |
| 16S-R | ccggacatgtcaaacgctgg |  |
| qRT-ndpR2F | ccgagctcatgaagcctgcg | To amplify the *ndpR2* for qRT-PCR |
| qRT-ndpR2R | cgctcgtgcggagactcaag |  |
| qRT-ndpRF | ggtcaagcgctacgtcg | To amplify the *ndpR* for qRT-PCR |
| qRT-ndpRR | gcatctcgtccttagacagc |  |
| Reporter R2F | cgcagtgatcgtacgacca | To construct reporter vector of P*_ndpR2_* |
| Reporter R2R | tccagccagaggccgttc |  |
| Reporter RF | atcgtatcagggacgccac | To construct reporter vector of P*_ndpR_* |
| Reporter RR | cgccttcactttctcggg |  |
| Reporter VF | gctgacgccgttggatacac | For the verification of the construction of reporter vectors of promoters by PCR or sequence |
| Reporter VR | tagatgggcgcatcgtaacc |  |
| Integration VF1F | ggcaataccgtttcggcatca | For the verification of the integration of reporter vectors in 5’ flanking site of the insertion sequence |
| Integration VF1R | attggtggcgacgactcctg |  |
| Integration VF2F | aaacagcattccaggtattaga | For the verification of the integration of reporter vectors in 3’ flanking site of the insertion sequence |
| Integration VF2R | tcttcccaagcgtccc |  |
| R2F0-F | cgcagtgatcgtacgacca | To amplify the F0 fragment of P*_ndpR2_* |
| R2F0-R | tccagccagaggccgttc |  |
| R2F1-F | cagccagaggccgttcgc | To amplify the F1 fragment of P*_ndpR2_* |
| R2F1-R | gagcgtggcgaacgagtc |  |
| R2F2-F | ttctgctcccatccacactg | To amplify the F2 fragment of P*_ndpR2_* |
| R2F2-R | ttcacatcccacgcgctc |  |
| R2F3-F | tcgccaacgactctccaatc | To amplify the F3 fragment of P*_ndpR2_* |
| R2F3-R | cgcagtgatcgtacgacca |  |
| R2Fa-F | ttctgctcccatccacactg | To amplify the Fa subfragment of P*_ndpR2_* |
| R2Fa-R | tcccctcgtccacggttc |  |
| R2Fb-F | tcgccaacgactctccaatc | To amplify the Fb subfragment of P*_ndpR2_* |
| R2Fb-R | ggtagggagaagccgttg |  |
| R2Fc-F | gagcgcgtgggatgtgaa | To amplify the Fc subfragment of P*_ndpR2_* |
| R2Fc-R | cgcagtgatcgtacgacca |  |
| FAM-M13F | 6FAM-caggaaacagctatgac | P*_ndpR2_* sequence obtained by PCR for DNase I footprinting assay |
| M13R | gttttcccagtcacgac |  |
| TetR-M-1F | atcagcatcgctgacatttcggaacgcgcc | To mutate ndpR (R55A) in the plasmid PUC19-*ndpR*-P*_ndpR2_*-*gfp* by inverse PCR |
| TetR-M-1R | aatgtcagcgatgctgatgccagcgtagc |  |
| TetR-M-2F | gacatttcggctcgcgccggcgtcaaccag | To mutate NdpR (E59A) in the plasmid PUC19-*ndpR*-P*_ndpR2_*-*gfp* by inverse PCR |
| TetR-M-2R | ggcgcgagccgaaatgtcgcggatgctgatgccag |  |
| TetR-M-4F | tcaaccaggctctgatccgctactatttcgagagcaagcagcagc | To mutate NdpR (G66A) in the plasmid PUC19-*ndpR*-P*_ndpR2_*-*gfp* by inverse PCR |
| TetR-M-4R | cggatcagagcctggttgacgccggcgcgtt |  |
| TetR-2M-34F | tcaacgctgctctgatccgctactatttcgagagcaagcag | To mutate NdpR (Q65A/G66A) in the plasmid PUC19-*ndpR*-P*_ndpR2_*-*gfp* by inverse PCR |
| TetR-2M-34R | cggatcagagcagcgttgacgccggcgcgtt |  |
| TetR-M-R | atgctgatgccagcgtagc | To mutate NdpR (R55A/E59A) in the plasmid PUC19-*ndpR*-P*_ndpR2_*-*gfp* by inverse PCR |
| TetR-2M-1F | ctacgctggcatcagcatcgctgacatttcggctcgcgccggcgtcaaccaggggctga |  |
| TetR-M-56F | tttcgagagcaagcagcagct | To mutate NdpR (Y70A) in the plasmid PUC19-*ndpR*-P*_ndpR2_*-*gfp* by inverse PCR |
| TetR-M-5R | ctgctgcttgctctcgaaatagtaagcgatcagcccctggttgacgc |  |
| TetR-M-5R | ctgctgcttgctctcgaaatagtaagcgatcagcccctggttgacgc | To mutate NdpR (R69A) in the plasmid PUC19-*ndpR*-P*_ndpR2_*-*gfp* by inverse PCR with TetR-M-56F primer |
| TetR-2M-56R | ctgctgcttgctctcgaaataagcagcgatcagcccctggttgacgc | To mutate NdpR (R69A/Y70A) in the plasmid PUC19-*ndpR*-P*_ndpR2_*-*gfp* by inverse PCR with TetR-M-56F primer |
| TetR-M-34R | cgttccgaaatgtcgcggat | To mutate NdpR (Q65A) in the plasmid PUC19-*ndpR*-P*_ndpR2_*-*gfp* by inverse PCR |
| TetR-M-3F | ccgcgacatttcggaacgcgccggcgtcaacgctgggctgat |  |
| TetR-4M-F | ccgcgacatttcggaacgcgccggcgtcaacgctgctctgatcgctgcttatttcgagagcaagcagcagc | To mutate NdpR (Q65A/G66A/R69A/Y70A) in the plasmid PUC19-*ndpR*-P*_ndpR2_*-*gfp* by inverse PCR with TetR-M-34R primer |
| TetR-M-7F | ccgctactatttcgagagcgctcagcagctgttcgacgaggtc | To mutate NdpR (K75A) in the plasmid PUC19-*ndpR*-P*_ndpR2_*-*gfp* by inverse PCR |
| TetR-M-7R | gctctcgaaatagtagcggat |  |
| NTetR-2M-34F | ccgcgacatttcggaacgcgccggcgtcaacgctgtcctgatccgctactatttcgagagc | To mutate NdpR (Q65A/G66V) in the plasmid PUC19-*ndpR*-P*_ndpR2_*-*gfp* by inverse PCR with TetR-M-34R primer |
| tetR-YZ-F | aggaggtgcaggagaagcc | For sequencing verification of NdpR mutation |
| tetR-YZ-R | gaggtaggtgccgacgagg |  |
| FX-pMM-F | ggcgctggattcccgttgcacatagcccg | pMM replicon and replication-associated protein sequences obtained by PCR |
| FX-pMM-R | cacttacggggcagcgctcagtaggcaa |  |
| pET28a-lacI-F | cacctcgacctgaatggatcactgcccgctttccag | lacI gene fragment obtained by PCR |
| pET28a-lacI-R | cacctcgacctgaatggatcactgcccgctttccag |  |
| pUC19-*ndpR*-F | cgatggtgtccctgattctgtggataaccgtattac | P*_lac_* promoter-controlled ndpR gene fragment obtained by PCR |
| pUC19-*ndpR-*R | aggaactatatccggtcgcgcgtttcggtgatga |  |
| FX-PMMH-F | ctgtcagaccaagtttactcatat | Linearized vector fragment with resistance gene to be replaced obtained by PCR |
| FX-PMMH-R | actcttcctttttcaatattattgaagc |  |
| Gm-F | aatattgaaaaaggaagagtatgttacgcagcagcaac | Gm resistance gene fragment obtained by PCR |
| Gm-R | agtaaacttggtctgacagttaggtggcggtacttggg |  |
| GFP Term-F | ccggatatagttcctcctttcagcaaaaaacccctcaagacccgtttagaggccccaaggggttatgctagttatt | Obtain the egfp gene fragment with a T7 terminator at its 3' end and a multiple cloning site (MCS) containing restriction enzyme sites added at its 5' end via primers by PCR. |
| GFP MCS-R | gaggccctttcgtcactagtacgtatgcatccatgggctagcgttaacaggcctctcgagtttgtttaactttaagaaggagatatacatatggtg |  |
| NdpR2 F | cttgttctgatcggaacgatcgttggctgtcaggatgccaatccgcc | Obtain the promoter and coding gene fragment of *ndpR2* by PCR. |
| NdpR2 R | ccacggtgtgcgtccatgcgtcacgttcgaactcgg |  |
| PndpA-F | gttaaacaaactcgagaggcaagaccatcatcgcggcga | Obtain the promoter P*_ndpA_* by PCR. |
| PndpA-R | gaggccctttcgtcactagatgaaccataagaaagcgctcct |  |
| PndpR2-F | gttaaacaaactcgagaggctaacggctcctctcttctgct | Obtain the promoter P*_ndpR2_* by PCR. |
| PndpR2-R | cgaggccctttcgtcactagcgcagtgatcgtacgaccaat |  |
| PMM YZ-F | ggtcagagtagtgacaagtgttg | Verify the constructed PMTF plasmid and promoter insertion by PCR and sequencing. |
| PMM YZ-R | gctgtgcctccatgatagcc |  |
| PMM YZ-R2 | cgaaaagtgccacctgacg |  |
| 142M1-F | cccgcctggctgccgagccagaggagcacgc | To mutate NdpR(H142A) in the plasmid pET28a-*ndpR* by inverse PCR |
| 142M1-R | ggctcggcagccaggcgggcctgaagcct |  |
| 153M2-F | tccgcctggctcgggaggtctacgacgcatcgg | To mutate NdpR(R153A) in the plasmid pET28a-*ndpR* by inverse PCR |
| 153M2-R | gacctcccgagccaggcggagcgcgtgct |  |
| 157M3-F | cgggaggtcgctgacgcatcggtcaagcgctacgt | To mutate NdpR(Y157A) in the plasmid pET28a-*ndpR* by inverse PCR |
| 157M3-R | gatgcgtcagcgacctcccgccgcaggc |  |
| 200M4-F | acctcggcgctatagaggatttgacagaaggcagg | To mutate NdpR(R200A) in the plasmid pET28a-*ndpR* by inverse PCR |
| 200M4-R | tcctctatagcgccgaggtcgttcagca |  |
| NdpR MYZ-F | cagccatatggctagcatg | For sequencing verification of NdpR mutation |
| NdpR MYZ-R | gttattgctcagcggtgg |  |

## Table S3. Structural and thermodynamic analysis of the NdpR dimerization interface.

| **Interface Summary** | | | | |
| --- | --- | --- | --- | --- |
|  | Structure 1 | | Structure 2 | |
| Selection range | A | | B | |
| **class** | Protein | | Protein | |
| **symmetry operation** | x,y,z | | ,, | |
| **symmetry ID** | 1_555 | | 0_555 | |
| Number of atoms |  |  |  |  |
| **interface** | 243 | 14.00% | 243 | 14.00% |
| **surface** | 1039 | 59.80% | 1013 | 58.30% |
| **total** | 1737 | 100.00% | 1737 | 100.00% |
| Number of residues |  |  |  |  |
| **interface** | 56 | 25.90% | 61 | 28.20% |
| **surface** | 200 | 92.60% | 205 | 94.90% |
| **total** | 216 | 100.00% | 216 | 100.00% |
| Solvent-accessible area, Å |  |  |  |  |
| **interface** | 2305 | 19.70% | 2403 | 20.30% |
| **total** | 11697.4 | 100.00% | 11813.2 | 100.00% |
| Solvation energy,kal/mol |  |  |  |  |
| **isolated structure** | -162.8 | 100.00% | -167.2 | 100.00% |
| **gain on complex formation** | -11 | 6.80% | -11.3 | 6.70% |
| **average gain** | -8.3 | 5.10% | -6.7 | 4.00% |
| **P-value** | 0.303 |  | 0.199 |  |

| **Salt bridges** | | | |
| --- | --- | --- | --- |
| **##** | structure 1 | Dist. [Å] | structure 2 |
| 1 | A:ARG 140[NH1] | 3.68 | B:GLU 144[OE1] |
| 2 | A:ARG 140[NH1] | 2.88 | B:GLU 144[OE2] |
| 3 | A:ARG 140[NH2] | 2.80 | B:GLU 144[OE2] |
| 4 | A:ARG 200[NH1] | 2.41 | B:ASP 158[OD1] |
| 5 | A:ARG 200[NH2] | 3.44 | B:ASP 158[OD1] |
| 6 | A:ARG 200[NH1] | 3.26 | B:ASP 158[OD2] |
| 7 | A:ARG 200[NH2] | 2.99 | B:ASP 158[OD2] |
| 8 | A:ARG 154[NH2] | 3.55 | B:ASP 203[OD1] |
| 9 | A:ARG 154[NH2] | 3.03 | B:ASP 203[OD2] |
| 10 | A:ARG 154[NE] | 2.63 | B:ASP 203[OD2] |
| 11 | A:ARG 177[NH2] | 3.42 | B:GLU 206[OE1] |
| 12 | A:LYS 162[NZ] | 2.19 | B:GLU 206[OE2] |
| 13 | A:ARG 177[NH2] | 3.14 | B:GLU 206[OE2] |
| 14 | A:GLU 144[OE1] | 3.55 | B:ARG 140[NH2] |
| 15 | A:GLU 144[OE2] | 2.47 | B:ARG 140[NH1] |
| 16 | A:GLU 144[OE2] | 2.40 | B:ARG 140[NH2] |
| 17 | A:ASP 158[OD1] | 2.90 | B:ARG 200[NH2] |
| 18 | A:ASP 158[OD1] | 2.62 | B:ARG 200[NH1] |
| 19 | A:ASP 158[OD2] | 2.64 | B:ARG 200[NH2] |
| 20 | A:ASP 158[OD2] | 3.37 | B:ARG 200[NH1] |
| 21 | A:ASP 203[OD1] | 3.09 | B:ARG 154[NE] |
| 22 | A:ASP 203[OD1] | 2.54 | B:ARG 154[NH2] |
| 23 | A:ASP 203[OD2] | 2.42 | B:ARG 154[NE] |
| 24 | A:ASP 203[OD2] | 3.37 | B:ARG 154[NH2] |
| 25 | A:GLU 206[OE1] | 3.17 | B:ARG 177[NH1] |
| 26 | A:GLU 206[OE1] | 2.73 | B:ARG 177[NH2] |
| 27 | A:GLU 206[OE1] | 3.47 | B:LYS 162[NZ] |
| 28 | A:GLU 206[OE2] | 3.63 | B:ARG 177[NH1] |

| **Hydrogen bonds** | | | |
| --- | --- | --- | --- |
| **##** | structure 1 | Dist. [Å] | structure 2 |
| 1 | A:ARG 140[NH1] | 2.88 | B:GLU 144[OE2] |
| 2 | A:ARG 140[NH2] | 2.80 | B:GLU 144[OE2] |
| 3 | A:ARG 200[NH1] | 2.79 | B:ARG 153[O] |
| 4 | A:ARG 200[NH1] | 2.41 | B:ASP 158[OD1] |
| 5 | A:ARG 200[NH2] | 2.99 | B:ASP 158[OD2] |
| 6 | A:ARG 153[NH2] | 3.05 | B:LEU 198[O] |
| 7 | A:ARG 154[HE] | 1.63 | B:ASP 203[OD2] |
| 8 | A:LYS 162[HZ2] | 1.33 | B:GLU 206[OE2] |
| 9 | A:ARG 177[NH2] | 3.14 | B:GLU 206[OE2] |
| 10 | A:GLU 144[OE2] | 2.40 | B:ARG 140[NH2] |
| 11 | A:ARG 153[O] | 2.33 | B:ARG 200[NH1] |
| 12 | A:ASP 158[OD1] | 2.62 | B:ARG 200[NH1] |
| 13 | A:ASP 158[OD2] | 2.64 | B:ARG 200[NH2] |
| 14 | A:LEU 198[O] | 3.50 | B:ARG 153[NH2] |
| 15 | A:ASP 203[OD1] | 2.54 | B:ARG 154[NH2] |
| 16 | A:LEU 204[O] | 3.62 | B:ARG 177[NH2] |
| 17 | A:GLU 206[OE1] | 2.73 | B:ARG 177[NH2] |
| 18 | A:ARG 208[O] | 3.65 | B:ARG 178[NH1] |

The interaction interface parameters were calculated using the PDBePISA server (Protein Interfaces, Surfaces and Assemblies). The interaction interface is primarily stabilized by a hydrogen bonding network involving 18 amino acid residues located on helices α8, α9, and α10, alongside salt bridges formed at 28 positions. The Δ^i^G value (-22.3 kcal/mol) represents the dimerization free energy change, indicating a favorable solvation free energy gain upon the formation of the interface. The corresponding Δ^i^G P-value of 0.245 (which is < 0.5) statistically supports that this interaction surface is highly specific for NdpR dimerization.

## References

1.	Guo K, Wang W, Wang H, Lu Z, Gao H. 2019. Complex oxidation of apocytochromes *c* during bacterial cytochrome *c* maturation. Appl Environ Microbiol 85:e01989-e01919.

2.	Xu Y, Guo R, Wang R, Su Z, Wu Y, Xie C, Wang P. 2025. Fumarate and nitrate reduction regulator (FNR) modulates hypermucoviscosity and virulence in hypervirulent *Klebsiella pneumoniae* through anaerobic adaptation. Virulence 16:2536186.

3.	Wang H, Tang Q, He Z, Hu T, Qiao P, Lu Z, Zhong W. 2025. The AraC/XylS family transcriptional regulator NdpR2 positively regulates nicotine catabolism in *Sphingomonas melonis* TY. Process Biochem 158:44-53.

4.	Wang H, Wang X, Tang Q, Wang L, Mei C, Shao Y, Xu Y, Lu Z, Zhong W. 2023. Regulation mechanism of nicotine catabolism in *Sphingomonas melonis* TY by a dual role transcriptional regulator NdpR. Appl Environ Microbiol 89:e0032423.

5.	Wang Y, Cen XF, Zhao GP, Wang J. 2012. Characterization of a new GlnR binding box in the promoter of *amtB* in *Streptomyces coelicolor* inferred a PhoP/GlnR competitive binding mechanism for transcriptional regulation of *amtB*. J Bacteriol 194:5237-44.

6.	Woodcock DM, Crowther PJ, Doherty J, Jefferson S, DeCruz E, Noyer-Weidner M, Smith SS, Michael MZ, Graham MW. 1989. Quantitative evaluation of Escherichia coli host strains for tolerance to cytosine methylation in plasmid and phage recombinants. Nucleic Acids Res 17:3469-3478.

7.	Cheng M, Xing Z, Lu L, Chen F, He J, Huang X. 2020. A plasmid-based genomic screening system for transcriptional regulators of non-adjacent xenobiotic catabolism genes. Appl Microbiol Biotechnol 104:1163-1174.
